# Does antipsychotic drug use increase the risk of long term mortality? A systematic review and meta-analysis of observational studies

**DOI:** 10.18632/oncotarget.24120

**Published:** 2018-01-10

**Authors:** Chunsong Yang, Zilong Hao, Jinhui Tian, Wei Zhang, Wenting Li, Ling-Li Zhang, Fujian Song

**Affiliations:** ^1^ Department of Pharmacy, Evidence-Based Pharmacy Center, West China Second Hospital, Sichuan University, Chengdu, China; ^2^ Key Laboratory of Birth Defects and Related Diseases of Women and Children, Sichuan University, Ministry of Education, Chengdu, China; ^3^ Department of Neurology, West China Hospital, Sichuan University, Chengdu, China; ^4^ Evidence-Based Medicine Center of Lanzhou University, Lanzhou, China; ^5^ Mental Health Center, West China Hospital of Sichuan University, Chengdu, China; ^6^ Norwich Medical School, Faculty of Medicine and Health Science, University of East Anglia, Norwich, Norfolk, UK

**Keywords:** antipsychotic drug, mortality, systematic review, meta-analysis

## Abstract

Antipsychotics (AP) are widely used to treat schizophrenia and other psychiatric disorders. However, the association between the AP use and mortality risk is controversial. We searched PubMed, EMBASE, MEDLINE, PsycINFO, CINAHL, the Cochrane Library and four Chinese databases from inception to June 2016. All observational cohort or case–control studies reporting data on mortality outcomes in individuals exposed to AP drugs were included. This systematic review included 68 studies involving 4,812,370 participants. Sixty-seven studies reported confounding factors, the most common being age, sex, race, concomitant medications, and comorbidities. For all-cause mortality, current users of AP and conventional antipsychotics (CAP) had higher mortality risk than did non-AP users [AP users: RR, 1.50; 95% CI, 1.12 to 1.99; CAP users: RR, 1.53; 95% CI, 1.16 to 2.04]. However, the association between the current use of atypical antipsychotics (AAP) and the mortality was of borderline significance, and there was no significant difference for past users of AP. Mortality was higher in current CAP users than in current AAP users. For cardiac death and sudden death, current AP and CAP users also had higher mortality risk than non-AP users. A subgroup analysis showed a possible increased risk in patients with Parkinson’s, but not in those with dementia, Alzheimer’s disease, schizophrenia, delirium or stroke. An increased risk of all-cause mortality for patients ≧65 years may also exist. AP exposure is associated with an approximately 1.5-fold increased mortality risk. This increased risk may be particularly prominent in patients with Parkinson’s and those over 65 years old. Further studies are required to evaluate the mortality risk for individual AP drugs and diseases.

## INTRODUCTION

Antipsychotics, including conventional antipsychotics (CAP) and atypical antipsychotics (AAP), are widely used to treat schizophrenia, behavioral and psychological symptoms in patients with dementia, and other psychotic disorders [1–3]. Up to 46% of elderly patients in nursing homes are treated with AP [4–7] and 90% of nursing home residents with dementia have at least one behavioral or psychological symptom, meaning patients are prescribed AP [8].

However, concerns about the safety of AP has always been a controversial topic. In 2005, Schneider et al. [9] reported AAP use was associated with a 1.5-fold increased risk of death compared with placebo in elderly people with dementia based on data from 15 randomized controlled trials (RCTs). However, Hulshof et al. [10] reported that CAP did not increase the risk of mortality in elderly patients. As these systematic reviews only included RCTs, they reflect the risk of mortality across the short term. Some systematic reviews have explored the association between AP use and risk of mortality by including up-to-date evidence from observational studies. Trifiro et al. [11] provided mixed results on the safety of AP to treat psychological symptoms of dementia. Zhai et al. [12] reported that AP did not increase the risk of death in patients with Alzheimer’s disease. However, the former systematic reviews [11] only described the results of included studies and lacked a quantitative analysis.

Current systematic reviews only focused on the mortality for the specific disease and the results were controversial, so if our study includes all types of research without limiting the type of disease, it will provide more information about the overall risk of death and some important outcomes. Moreover, one systematic review [12] already reported a lot of heterogeneity in study results, but it did not well explain the source of heterogeneity, we will use the method of meta regression to study potential explanations more explicitly and more extensively than they did in the current analysis. In addition, several observational studies have been recently published that investigate the risk of mortality associated with AP use. Therefore, we conducted an updated systematic review of observational studies by combining all available data to derive an estimation of the association between AP use and long term risk of mortality in real world clinical practice.

## RESULTS

### Results of the literature search

**Figure 1:**
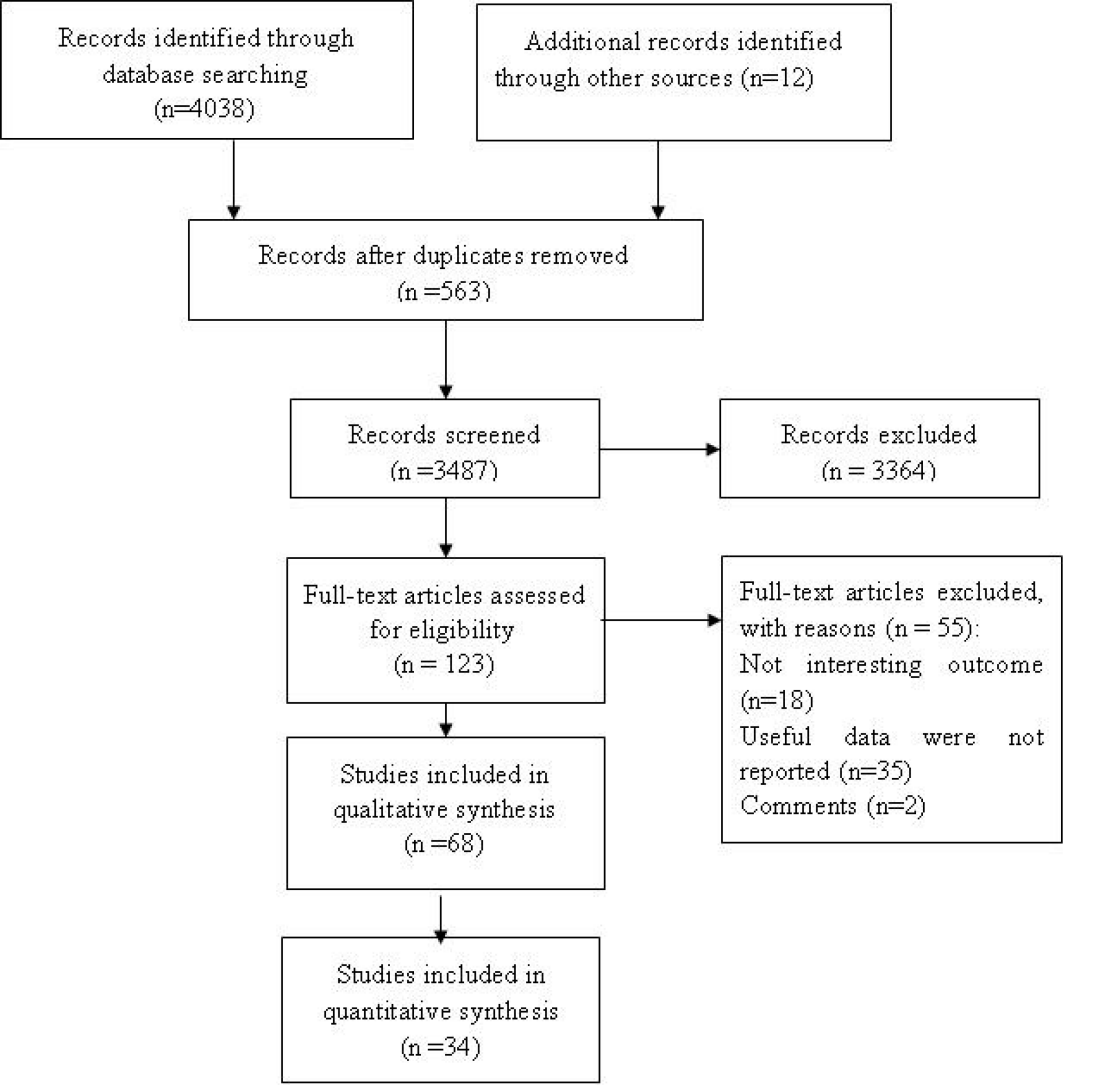
Flowchart of the search strategy

Initially, we identified 4498 articles. After removing duplicates, screening titles and abstracts, and reading full texts, 68 observational studies (*n* = 4,812,370) met the inclusion criteria, including 51 cohort studies (*n* = 2,033,567) and 17 case-control studies (*n* = 2,778,803) (Figure 1).

### Study characteristics

Supplementary Table 1 presents the characteristics of included studies. We included 68 studies involving 4,812,370 participants, with sample sizes varying from 153 to 1,716,552 (median 5,949). The residence of the first author varied as follows: Europe (35/68, 51.5%), North America (25/68, 36.8%), Oceania (4/68, 5.9%), and Asia (4/68, 5.9%).

The age of participants ranged from 10 to 100 years. Forty-nine studies reported a follow up period ranging from 30 days to 16 years, with the following distribution: 3 months or less (7/49, 14.3%), 6 months (15/49, 30.6%), 1 year (4/49, 8.2%), 1–5 years (17/49, 34.7%), and >5 years (6/49, 12.2%). Sixty-seven studies reported confounding factors, the most common being age, sex, race, concomitant medications, and comorbidities.

### Quality assessment (Supplementary Table 2)

Of the 68 studies, 66 (97.1%) were rated as high quality (scoring 8.04 ± 0.69), 2 (2.9%) were of medium quality (scoring 6), and there were no low quality studies. A maximum score for the Newcastle-Ottawa Scale was achieved by 17 studies (25%). All studies gained the maximum score in the selection outcome, 37 studies had the maximum score in the comparability outcome, and 33 studies had the maximum score in the exposure outcome.

### Antipsychotic drugs and mortality risk

#### AP users vs. AP non-users

**Figure 2:**
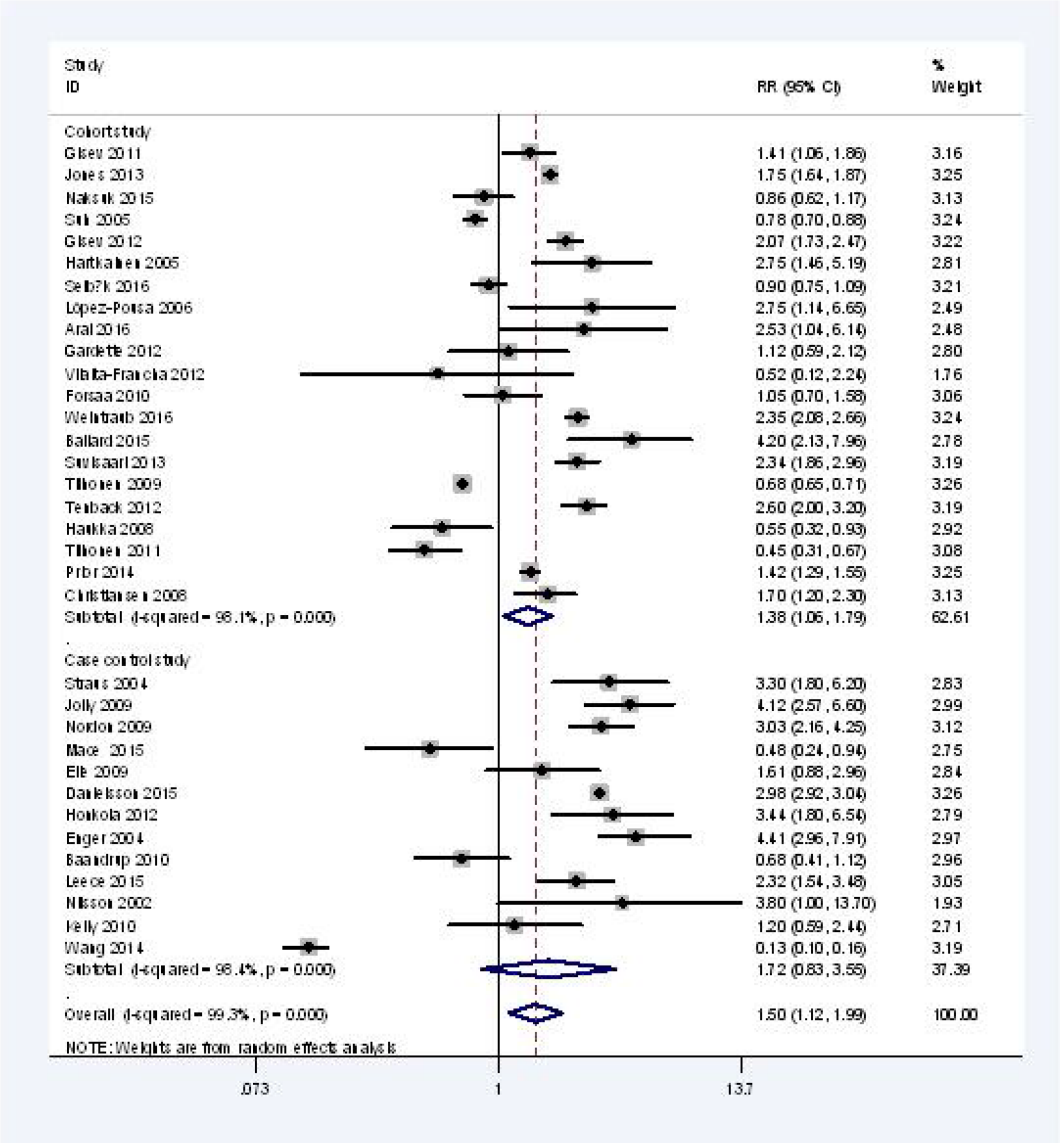
All-cause mortality of current AP user vs AP non-users

All-cause mortality risk was reported by 34 studies (*n* = 2,500,546), including 21 cohort studies (*n* = 679,683) and 13 case-control studies (*n* = 1,820,863). The pooled RR for 34 observational studies was 1.50 [95% CI, 1.12 to 1.99; I^2^, 99.3%] for current AP users compared with those not exposed to AP (Figure 2).

Seven studies (*n* = 409,294), including two cohort studies (*n* = 384,299) and five case-control studies (*n* = 24,995) reported cardiac death risks for patients prescribed AP, and the risk for current AP use was higher than that for AP non-users [RR, 2.10; 95% CI, 1.29 to 3.42; I^2^, 76.7%].

Only three case-control studies (*n* = 11,789) reported the outcome of sudden death. Our meta-analysis showed that the risk of sudden death for current AP use was higher than that for AP non-users [RR, 3.70; 95% CI, 2.68 to 5.12; I^2^, 0%] (Figure not shown).

### Current CAP users vs. AP non-users

All-cause mortality risk for CAP users was reported in 20 studies (*n* = 856,461), including 14 cohort studies (n = 788,566) and 6 case-control studies (*n* = 67,895). The risk for current CAP users was higher than that for AP non-users [RR, 1.53; 95% CI, 1.16 to 2.04, I^2^, 96.6%].

Cardiac death was reported in two studies (*n* = 384,299) and sudden death was also reported in two studies (*n* = 184,046). Current CAP users had higher rates of cardiac death and sudden death compared with AP non-users [RR, 1.90; 95% CI, 1.52 to 2.30; I^2^, 0%; RR, 2.60; 95% CI, 1.01 to 6.74; I^2^, 65.6%, respectively] (Figure not shown).

### Current AAP users vs. AP non-users

All-cause mortality risk for current AAP users was reported in 26 studies (*n* = 1,080,378), including 19 cohort studies (*n* = 978,576) and 7 case-control studies (*n* = 101,802). The results showed the risk for current AAP users did not differ significantly from that for AP non-users [RR, 1.31; 95% CI, 1.00 to 1.72; I^2^, 98%].

In studies of AAP use, two studies (*n* = 384,299) reported the outcome of cardiac death and two studies (*n* = 186,625) reported the outcome of sudden death. The results revealed that, compared with AP non-users, current AAP users did not have higher rates of cardiac death [RR, 1.23; 95% CI, 0.57 to 2.65; I^2^, 84.8%], but did have higher rates of sudden death [RR, 1.94; 95% CI, 1.58 to 2.39; I^2^, 0%] (Figure not shown).

### Current CAP users vs. AAP users

**Figure 3:**
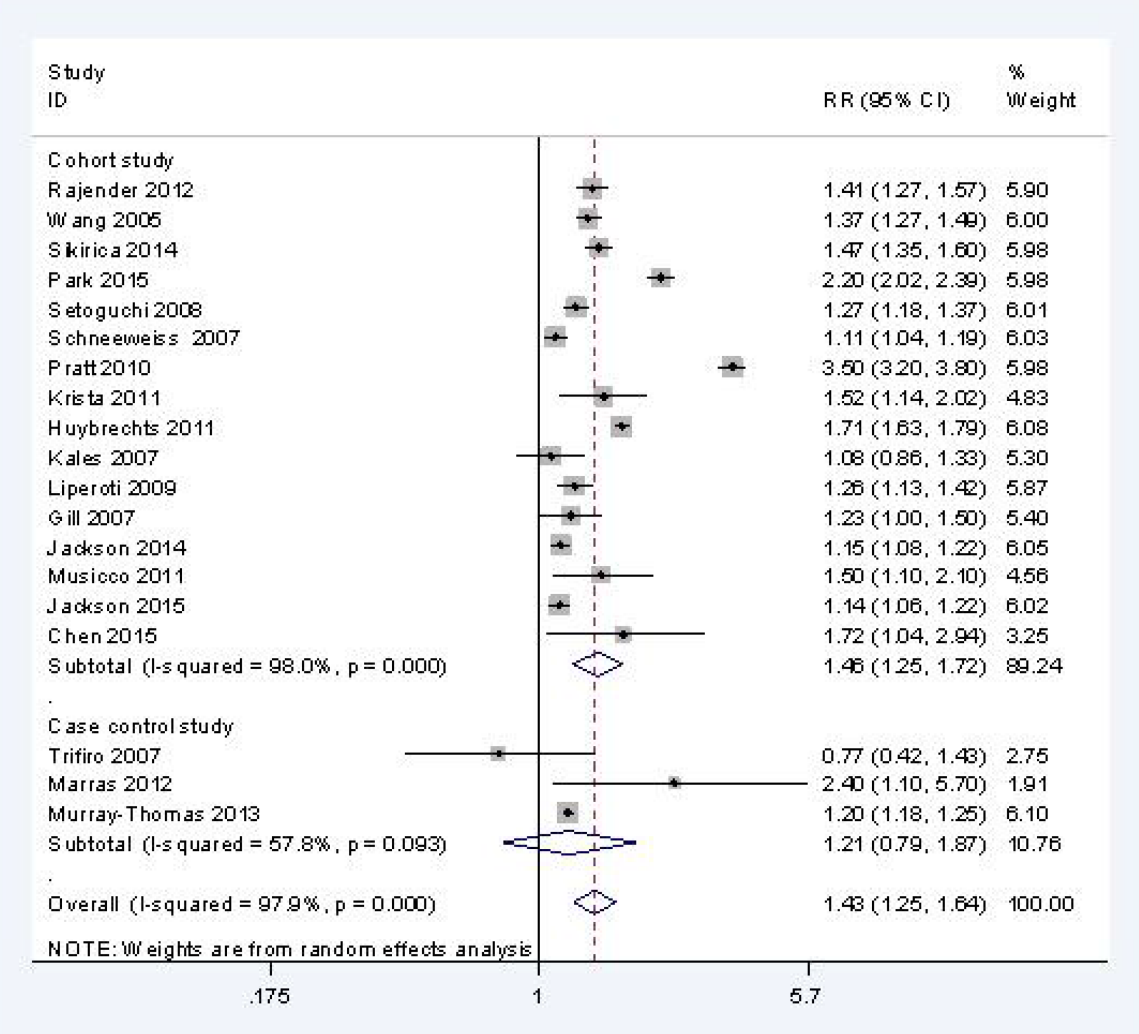
All cause mortality of current CAP users vs. AAP users

All-cause mortality in CAP and AAP users was reported in 19 studies (*n* = 1,381,688), including 16 cohort studies (*n* = 423,901) and three case control studies (*n* = 957,787). The results suggested that there was a higher risk all-cause mortality in current CAP users than in current AAP users [RR, 1.43; 95% CI, 1.25 to 1.64; I^2^, 97.9%] (Figure 3).

Cardiac death in CAP and AAP users was reported by two studies (*n* = 959,279) and sudden death in CAP and AAP users was reported by one study (*n* = 1,624). The results showed that the risk of both for current CAP users was higher than that for AAP users [RR, 1.17; 95% CI, 1.07 to 1.28; I^2^, 45.4%; RR, 1.72; 95% CI, 1.02 to 2.89; respectively] (Figure not shown).

### Past AP users vs. AP non- users

All-cause mortality risk for past AP use was reported in seven studies (*n* = 765,520), including six cohort studies (*n* = 760,503) and one case-control study (*n* = 5,017). The results showed the risk for past AP use did not differ significantly from that for AP non-use [RR, 1.01; 95% CI, 0.91 to 1.11; I^2^, 0%] (Supplementary Figure 1).

Cardiac death in past AP use was reported in three studies (*n* = 670,654). The results showed that the risk for past AP use did not differ significantly from that of AP non-use [RR, 1.02; 95% CI, 0.85 to 1.21; I^2^, 24.6%] (Figure not shown).

### Subgroup analyses

#### Mortality among different diagnoses

**Figure 4:**
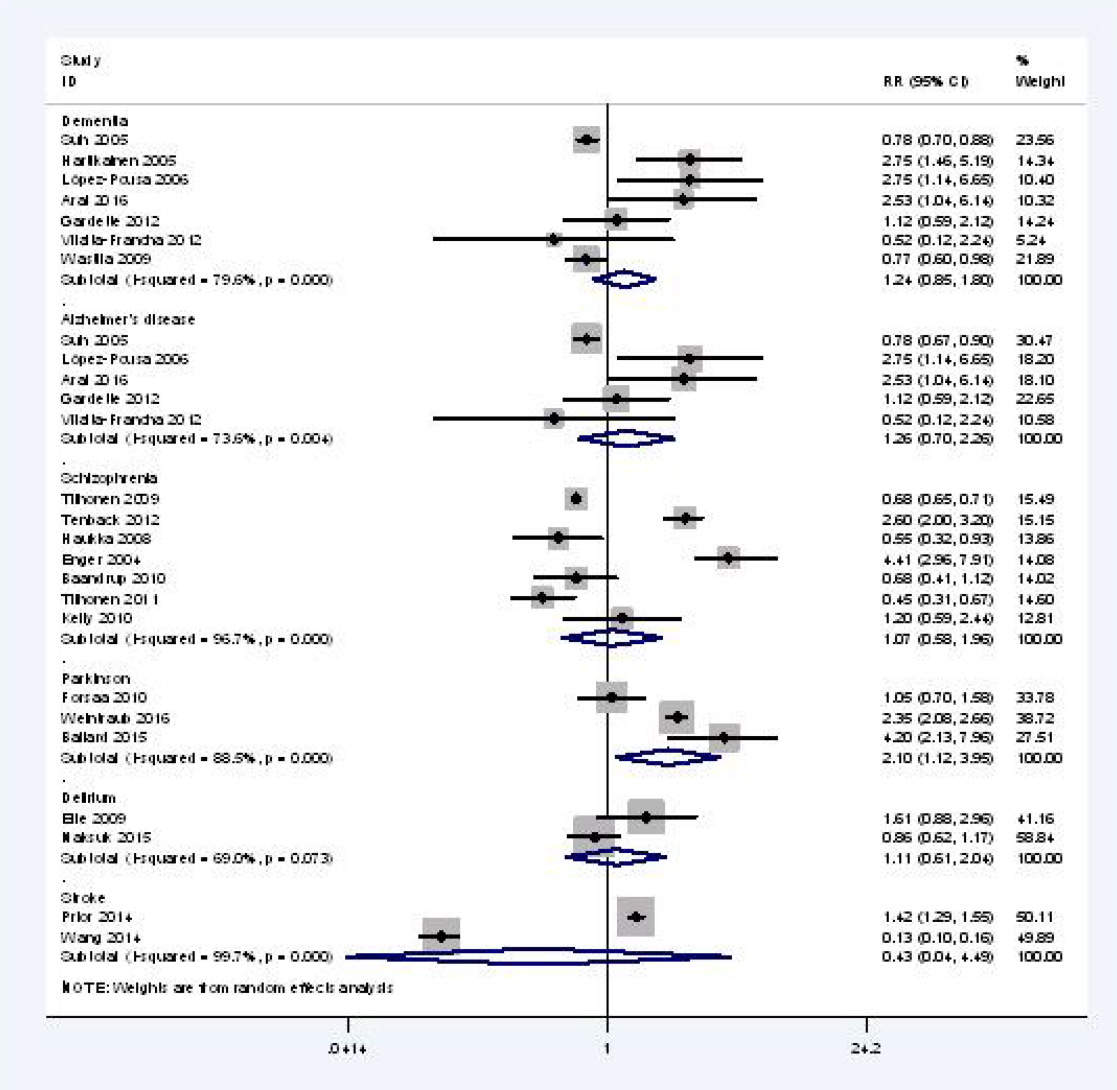
All cause mortality of current AP user vs AP non-users for different disease

In the subgroup analyses for mortality in patients with different diseases (Figure 4), our meta-analysis showed that, compared with AP non-users, only patients with Parkinson’s (three studies, *n* = 16,407) have higher all-cause mortality associated with AP use [RR, 2.10; 95% CI, 1.12 to 3.95; I^2^, 88.5%]. Although current AP users with dementia (seven studies, *n* = 12,655), AD (five studies, *n* = 11,781), schizophrenia (seven studies, *n* = 191,557), and delirium (two studies, *n* = 3,360) had an increased mortality risk, but the differences were not significant.

### Mortality according to age

In the subgroup analyses for mortality in patients of different ages (Supplementary Figure 2), compared with AP non-users, AP use increased the risk of all-cause mortality in patients over 65 years old (21 studies, *n* = 1,909,183), but not in patients under 65 years old (seven studies, *n* = 403,725) [RR, 1.59; 95% CI, 1.13 to 2.23; I^2^, 98.7%; RR, 1.09; 95% CI, 0.67 to 1.78; I^2^, 94.6%, respectively].

### Meta-regression

While conducting our meta-analysis of the risk of all-cause mortality in AP users compared with AP non-users, we observed high heterogeneity among the studies (I^2^, 99.3%). Therefore, we conducted a meta-regression using the covariates of study design, age, disease, and comorbidities. The results showed that none of these variables had a significant impact on between-study heterogeneity (*P* values, 0.889; 0.295; 0.173; and 0.140, 95% CI, -0.67-0.58, -0.22-0.70, -0.05-0.27,-0.06-0.44, respectively).

### Sensitivity analyses

In a sensitivity analysis that excluded one study at a time, the pooled RR for all-cause mortality in patients ranged from 1.45 (95% CI, 1.08 to 1.94) to 1.62 (95% CI, 1.23 to 2.14) (Supplementary Figure 3). All point estimates lay within the 95% CI of the combined analysis, indicating that no individual study had excessive influence on the pooled effect between AP use and all-cause mortality risk.

### Publication bias

Supplementary Figure 4 shows the results of our analysis of publication bias. Visual inspection of the funnel plot showed it to be asymmetrical. Egger’s test also showed no evidence of publication bias for all-cause mortality (*P* = 0.095).

## DISCUSSION

### Statement of main findings

In our study, the existing evidence indicated that current AP users have an approximately 1.5-fold increased risk of mortality than AP non-users. There were no significant differences in mortality risk for current AAP users and past AP users compared with AP non-users. However, the mortality risk of current CAP users was higher than that for current AAP users. Moreover, current CAP users also had higher rates of cardiac death and sudden death than AP non-users. We also found that an increased mortality risk may exist in AP users diagnosed with Parkinson’s but not in those with dementia, AD, schizophrenia, delirium, or stroke. There may also be an increased risk of all-cause mortality in patients over 65 years old. Although we have try to included all kinds of studies, the asymmetrical funnel plot indicated publication bias, the reason may be that some conference papers and papers published in other languages could not be included through our search.

### Quality of the evidence

In general, the studies included in this systematic review were of good quality. However, some problems did exist. First, in the exposure outcome, some studies did not control for important confounding factors (i.e., age, comorbidities, or concomitant medications). Second, the follow-up period in some cohort studies was short, so the outcome of interest might not have occurred during the period of observation. Third, although loss to follow up is common in cohort studies, few studies tested the comparability for the reason and proportion of loss of follow up between the exposure group and control group.

### Comparison with other studies

Several systematic reviews have investigated the association between AP use and risk of mortality. However, all previous systematic reviews have only focused on specific diseases. Schneider’s study [9] reported that CAP use carries a 1.5 times higher incidence of mortality in elderly people with dementia for relatively brief periods of less than 8 to 12 weeks, because it included six published trials and nine unpublished trial and all but one of the trials were sponsored and conducted by drug manufacturers. The likely reasons for the delays in publication were that most did not show statistically significant results on their primary efficacy outcomes, perhaps lessening the enthusiasm of the sponsors to submit the manuscripts. Moreover, death did not differ between the 6 published trials (OR, 1.42; 95% CI, 0.80-2.51) and the 9 unpublished trials (OR, 1.63; 95% CI, 1.00-2.65). Therefore the funnel plot of the log Ors against sample size was symmetrical around the mean overall effect, thus not providing evidence for selection bias in schneider’s study, but in our study, visual inspection of the funnel plot showed it to be asymmetrical and publication bias may exist. These were the most important reasons for the difference between the results of this study and our findings. Trifiro et al. [11] conducted an updated review of observational studies to examine the existing literature on the safety of antipsychotic drug use in dementia patients and found there is indeed a difference between the risks associated with individual antipsychotic drugs in psychological symptoms of dementia. In our review, we also found a similar increased risk among AP use over the long term. Another systematic review [12] focused on the mortality risk of patients with Alzheimer’s disease prescribed AP, reporting that AP use may not increase the death risk for these patients. This is consistent with our findings, as we also did not find an increased mortality risk for patients with Alzheimer’s disease. However, we found an increased risk of mortality in patients with Parkinson’s and those aged over 65 years old. In addition, we found that past AP use did not have an increased risk of mortality.

### Limitations

There are some limitations to our study. (1) Only studies published in English were included, and thus some non-English studies may have been missed. However, most high quality research is generally published in English journals. (2) Although statistical analyses were adjusted for several confounding factors, other potential confounding factors (i.e., genetic factors, race, comorbidity, follow-up duration) could not be entirely ruled out. (3) There was limited information about the dosages of AP exposure, so we could not conduct a dose-response analysis to assess the influence of different doses on mortality. Therefore, further studies should be conducted to overcome these shortcomings.

### Implications for future study

First, there was large heterogeneity when pooling the data from included studies. Even if most included studies had adjusted for potential confounding factors, some clinical differences in patients (i.e., age, disease, the setting of participants, comorbidities) could lead to clinical heterogeneity. Therefore, future research should focus on patients with specific diseases and ages to reduce clinical heterogeneity. Second, other potential confounding factors should be investigated. Third, we found about half of all studies had a follow up within one year, and only 12% studies had a follow up period of more than five years. Therefore, suitable follow up durations should be determined to demonstrate the risk of mortality in AP use. Lastly, it is necessary to establish a global multi-center registration platform to observe the risk of death over the long term.

## MATERIALS AND METHODS

### Inclusion and exclusion criteria

#### Types of studies

We included observational cohort and case–control studies published in English that reported data on mortality outcomes. Studies were excluded if (1) the outcomes of interest were not reported; (2) the effect size (i.e., odds ratio (OR), relative risk (RR) or hazard risk (HR) with 95% confidence intervals (CI) was not provided, or if these values could not be calculated from the data provided.

### Types of participants

Patients exposed to AP were included. There was no restriction with respect to indications for AP use (i.e., schizophrenia, behavioral and psychotic disturbances in dementia, delirium, stroke and Parkinson’s).

### Types of exposure and comparisons

The exposure of interest was AP use, including both CAP and AAP. The comparisons were as follows: (1) AP use vs. AP non-use; (2) CAP use vs. AP non-use; (3) AAP use vs. AP non-use; (4) AAP use vs. CAP use; (5) past AP use vs. AP non-use.

### Types of outcome measurements

The outcomes of interest were all-cause mortality, cardiac death, and sudden death. If there were different periods of follow up, we used the data from the last follow up.

### Search strategy

Two reviewers independently identified studies through searches of PubMed, EMBASE, MEDLINE, PsycINFO, CINAHL, the Cochrane Library and the Chinese Biomedical Literature Database, the China Knowledge Resource Integrated Database, the VIP Database, and the Wanfang Database from inception to June 2016, with no limits on date/time, language, document type, or publication status. We also searched for additional studies in the reference lists of all identified publications, including relevant meta-analyses and systematic reviews. Keywords were identified using experts’ opinion, literature review, controlled vocabulary (Medical Subject Headings = MeSH, Excerpta Medica Tree = EMTREE, APA Thesaurus, and CINHAL Headings), and reviewing the primary search results. Search strategies are reported in the Supplement.

### Selection of studies and data extraction

Two reviewers independently screened the titles and abstracts of every record. Full articles were obtained when either information provided in the title or the abstract conformed to the selection criteria outlined above, or when inclusion eligibility could not be ascertained because of limited information. For included studies, data were independently extracted by each reviewer and entered into a standardized form. The data extraction form included the following: (1) general study characteristics; (2) general patient characteristics; (3) study design; (4) sample size; (5) exposures and comparisons; and (6) outcomes of interest with effect size and 95% CI. Discrepancies were resolved by consensus.

### Quality assessment

Two reviewers independently evaluated the methodological quality of identified studies. The quality of observational studies was assessed using the Newcastle–Ottawa quality assessment scale (http://www.ohri.ca/programs/clinical_epidemiology/oxford.asp) as recommended by the Cochrane Collaboration [13]. The maximum score on the Newcastle–Ottawa quality assessment scale is 9. In our review, a score of 7–9 was considered as high quality, 4–6 as moderate quality, and 0–3 as low quality. Disagreements were discussed and agreed upon through consensus [14].

### Statistical methods

The outcome of interest was the effect of AP on the incidence of all-cause death, expressed as an OR, RR, or HR with 95% CIs. RRs were used as the common measure of association across studies. To do this, the HR was directly considered as an RR [15–17]. Death is a rare event, so the OR is approximately equal to RR. Therefore, an overall estimate was obtained from a random-effects model.

Subgroup analysis was performed according to the type of study (i.e., cohort study, case-control study), patient population (i.e., dementia, schizophrenia, Parkinson’s), and age (age ≥65 years vs. age <65 years). Meta-regression analyses were used to investigate whether potential heterogeneity could be explained. We will conduct a meta-regression using the covariates of study design, age, disease, and comorbidities. Sensitivity analyses were conducted to evaluate the stability of results by excluding one study at a time. Publication bias was assessed using visual inspection of funnel plots and Egger’s test.

Meta analyses were performed and presented using Stata 12.0 (Stata Corporation, College Station, Texas, USA).

## CONCLUSIONS

AP exposure is associated with an approximately 1.5-fold increased risk of mortality. This increased risk may be seen particularly in patients with Parkinson’s and those over 65 years old. Because of large heterogeneity in available studies, further research investigated on the risk of mortality for individual AP and different diseases is a priority.

## SUPPLEMENTARY MATERIALS FIGURES AND TABLES







## Abbreviations

AP: Antipsychotics
CAP: Conventional antipsychotics
AAP: Atypical antipsychotics
RCTs: Randomized controlled trials
OR: Odds ratio
RR: Relative risk
HR: Hazard risk
CI: Confidence intervals
