# Supplementary material for: Does antipsychotic drug use increase the risk of long term mortality? A systematic review and meta-analysis of observational studies
Source: Oncotarget. 2018 Jan 10;9(19):15101–10. doi: 10.18632/oncotarget.24120 (PMC5871101; doi:10.18632/oncotarget.24120)
Supplement: Supplementary file 1 [file oncotarget-09-15101-s001.pdf]

Does antipsychotic drug use increase the risk of long term mortality? A systematic review and meta-analysis of observational studies

SUPPLEMENTARY MATERIALS

A. CINAHL

S4 S1 AND S2 AND S3 Limiters-Exclude MEDLINE records

S3:(((MH “Randomized Controlled Trials”) OR (MH “Crossover Design”) OR (MH “Random Assignment”) OR (MH “Triple-Blind Studies”) OR (MH “Double-Blind Studies”) OR (MH “Single-Blind Studies”)) OR TI (Random* OR Crossover OR “Cross Over” OR ((Double OR Single OR Triple) N1 Blind*) OR (Control* N2 Trial*)) OR AB (Random* OR Crossover OR “Cross Over” OR ((Double OR Single OR Triple) N1 Blind*) OR (Control* N2 Trial*))) OR ((MH “Case Control Studies+”) OR (MH “Concurrent Prospective Studies”) OR (MH “Prospective Studies”) OR (MH “Observational Methods”) OR (MH “Nonexperimental Studies”)) OR TI (((Cohort OR Concurrent OR “Follow Up” OR Followup OR “Incidence Study” OR “Incidence Studies” OR Longitudinal OR Prospective OR Retrospective OR “Case Control” OR Observational OR “Self Controlled” OR “Case Series”)) OR AB (((Cohort OR Concurrent OR “Follow Up” OR Followup OR “Incidence Study” OR “Incidence Studies” OR Longitudinal OR Prospective OR Retrospective OR “Case Control” OR Observational OR “Self Controlled” OR “Case Series”))

S2:(MH “Antipsychotic Agents+”) OR TI (Acepromazineh or Aceprometazine or Amoxapine or Amperozide or Aripiprazole or Asenapine or Azaperone or Benperidol or Bifeprunox or Bromperidol or Butaclamol or Cariprazine or Chlorpromazine or Chlorprothixene or Clopenthixol or Clothiapine or Clozapine or Dapiprazole or Dicarbine or Dihydrohaloperidol or Dixyrazine or Droperidol or Ecopipam or Etazolate or Fananserin or Fencamfamine or Fluanisone or Flupenthixol or Fluperlapine or Fluphenazine or Fluspirilene or Haloperidol or Iloperidone or Isofloxythepin or Lenperone or Loxapine or Lurasidone or Mafoprazine or Mesoridazine or Methiothepin or Methotrimeprazine or Metylperon or Molindone or Nemonapride or Olanzapine or Ondansetron or Oxypertine or Oxyprothepine or Paliperidone or Penfluridol or Perazine or Perospirone or Perphenazine or Piflutixol or Pimozide or Pipamperone or Prochlorperazine or Promazine or Quetiapine or Raclopride or Remoxipride or Reserpine or Rimcazole or Risperidone or Ritanserin or Sertindole or Spiperone or Stepholidine or Sulforidazine or Sulpiride or Sultopride or Tetrabenazine or Tetrahydropalmatine or Thioridazine or Thiothixene or “Tiapride Hydrochloride” or Timiperone or Trifluoperazine or Trifluperidol or Triflupromazine or Vegetamin or Veralipride or Zetidoline or Ziprasidone or Zotepine or Antipsychotic* or Neuroleptic*) OR AB (Acepromazineh or Aceprometazine or Amoxapine or Amperozide or Aripiprazole or Asenapine or Azaperone or Benperidol or Bifeprunox or Bromperidol or Butaclamol or Cariprazine or Chlorpromazine or Chlorprothixene or Clopenthixol or Clothiapine or Clozapine or Dapiprazole or Dicarbine or Dihydrohaloperidol or Dixyrazine or Droperidol or Ecopipam or Etazolate or Fananserin or Fencamfamine or Fluanisone or Flupenthixol or Fluperlapine or Fluphenazine or Fluspirilene or Haloperidol or Iloperidone or Isofloxythepin or Lenperone or Loxapine or Lurasidone or Mafoprazine or Mesoridazine or Methiothepin or Methotrimeprazine or Metylperon or Molindone or Nemonapride or Olanzapine or Ondansetron or Oxypertine or Oxyprothepine or Paliperidone or Penfluridol or Perazine or Perospirone or Perphenazine or Piflutixol or Pimozide or Pipamperone or Prochlorperazine or Promazine or Quetiapine or Raclopride or Remoxipride or Reserpine or Rimcazole or Risperidone or Ritanserin or Sertindole or Spiperone or Stepholidine or Sulforidazine or Sulpiride or Sultopride or Tetrabenazine or Tetrahydropalmatine or Thioridazine or Thiothixene or “Tiapride Hydrochloride” or Timiperone or Trifluoperazine or Trifluperidol or Triflupromazine or Vegetamin or Veralipride or Zetidoline or Ziprasidone or Zotepine or Antipsychotic* or Neuroleptic*)

S1:((MH “Mortality”) OR (MH “Cause of Death”) OR (MH “Death”)) OR TI (Mortalit* OR Fatality OR Death) OR AB (Mortalit* OR Fatality OR Death)

B. COCHRANE LIBRARY

#1:MeSH descriptor: [Mortality] this term only; #2:MeSH descriptor: [Death] this term only; #3:MeSH descriptor: [Cause of Death] explode all trees; #4:(Mortalit* or Fatality or Death):ti,ab,kw; #5:#1 or #2

or #3 or #4; #6:MeSH descriptor: [Antipsychotic Agents] this term only; #7:MeSH descriptor: [Acepromazine] explode all trees; #8:MeSH descriptor: [Amoxapine] this term only; #9:MeSH descriptor: [Aripiprazole] this term only; #10:MeSH descriptor: [Azaperone] this term only; #11:MeSH descriptor: [Benperidol] this term only; #12:MeSH descriptor: [Butaclamol] this term only; #13:MeSH descriptor: [Chlorpromazine] this term only; #14:MeSH descriptor: [Chlorprothixene] this term only; #15:MeSH descriptor: [Clopenthixol] this term only; #16:MeSH descriptor: [Clozapine] this term only; #17:MeSH descriptor: [Droperidol] this term only; #18:MeSH descriptor: [Etazolate] this term only; #19:MeSH descriptor: [Flupenthixol] this term only; #20:MeSH descriptor: [Fluphenazine] this term only; #21:MeSH descriptor: [Fluspirilene] this term only; #22:MeSH descriptor: [Haloperidol] this term only; #23:MeSH descriptor: [Loxapine] explode all trees; #24:MeSH descriptor: [Mesoridazine] this term only; #25:MeSH descriptor: [Methiothepin] this term only; #26:MeSH descriptor: [Methotrimeprazine] this term only; #27:MeSH descriptor: [Molindone] this term only; #28:MeSH descriptor: [Ondansetron] this term only; #29:MeSH descriptor: [Paliperidone Palmitate] this term only; #30:MeSH descriptor: [Penfluridol] this term only; #31:MeSH descriptor: [Perazine] this term only; #32:MeSH descriptor: [Perphenazine] explode all trees; #33:MeSH descriptor: [Pimozide] this term only; #34:MeSH descriptor: [Prochlorperazine] this term only; #35:MeSH descriptor: [Promazine] this term only; #36:MeSH descriptor: [Quetiapine Fumarate] this term only; #37:MeSH descriptor: [Raclopride] this term only; #38:MeSH descriptor: [Remoxipride] this term only; #39:MeSH descriptor: [Reserpine] this term only; #40:MeSH descriptor: [Risperidone] this term only; #41:MeSH descriptor: [Ritanserin] this term only; #42:MeSH descriptor: [Spiperone] this term only; #43:MeSH descriptor: [Sulpiride] this term only; #44:MeSH descriptor: [Tetrabenazine] this term only; #45:MeSH descriptor: [Thioridazine] this term only; #46:MeSH descriptor: [Thiothixene] this term only; #47:MeSH descriptor: [Tiapride Hydrochloride] this term only; #48:MeSH descriptor: [Trifluoperazine] this term only; #49:MeSH descriptor: [Trifluperidol] this term only; #50:MeSH descriptor: [Triflupromazine] this term only; #51:(Acepromazineh or Aceprometazine or Amoxapine or Amperozide or Aripiprazole or Asenapine or Azaperone or Benperidol or Bifeprunox or Bromperidol or Butaclamol or Cariprazine or Chlorpromazine or Chlorprothixene or Clopenthixol or Clothiapine or Clozapine or Dapiprazole or Dicarbine or Dihydrohaloperidol or Dixyrazine or Droperidol or Ecopipam or Etazolate or Fananserin or Fencamfamine or Fluanisone or Flupenthixol or Fluperlapine or Fluphenazine or Fluspirilene or Haloperidol or Iloperidone or Isofloxythepin or Lenperone or Loxapine or Lurasidone or Mafoprazine or Mesoridazine or Methiothepin or Methotrimeprazine or Metylperon or Molindone or Nemonapride or Olanzapine or Ondansetron or Oxypertine or Oxyprothepine or Paliperidone or Penfluridol or Perazine or Perospirone or Perphenazine or Piflutixol or Pimozide or Pipamperone or Prochlorperazine or Promazine or Quetiapine or Raclopride or Remoxipride or Reserpine or Rimcazole or Risperidone or Ritanserin or Sertindole or Spiperone or Stepholidine or Sulforidazine or Sulpiride or Sultopride or Tetrabenazine or Tetrahydropalmatine or Thioridazine or Thiothixene or “Tiapride Hydrochloride” or Timiperone or Trifluoperazine or Trifluperidol or Triflupromazine or Vegetamin or Veralipride or Zetidoline or Ziprasidone or Zotepine or Antipsychotic* or Neuroleptic*):ti,ab; #52:#6 or #7 or #8 or #9 or #10 or #11 or #12 or #13 or #14 or #15 or #16 or #17 or #18 or #19 or #20 or #21 or #22 or #23 or #24 or #25 or #26 or #27 or #28 or #29 or #30 or #31 or #32 or #33 or #34 or #35 or #36 or #37 or #38 or #39 or #40 or #41 or #42 or #43 or #44 or #45 or #46 or #47 or #48 or #49 or #50 or #51; #53:#5 and #52.

C. EMBASE 1974 TO 2016 WEEK 25

1. Mortality/ OR Death/ OR Cause of Death/ OR (Mortalit* OR Fatality OR Death).af.

2. Exp Neuroleptic Agent/ OR (Acepromazineh OR Aceprometazine OR Amoxapine OR Amperozide OR Aripiprazole OR Asenapine OR Azaperone OR Benperidol OR Bifeprunox OR Bromperidol OR Butaclamol OR Cariprazine OR Chlorpromazine OR Chlorprothixene OR Clopenthixol OR Clothiapine OR Clozapine OR Dapiprazole OR Dicarbine OR Dihydrohaloperidol OR Dixyrazine OR Droperidol OR Ecopipam OR Etazolate OR Fananserin OR Fencamfamine OR Fluanisone OR Flupenthixol OR Fluperlapine OR Fluphenazine OR Fluspirilene OR Haloperidol OR Iloperidone OR Isofloxythepin OR Lenperone OR Loxapine OR Lurasidone OR Mafoprazine OR Mesoridazine OR Methiothepin OR Methotrimeprazine OR Metylperon OR Molindone OR Nemonapride OR Olanzapine OR Ondansetron OR Oxypertine OR Oxyprothepine OR Paliperidone OR Penfluridol OR Perazine OR Perospirone OR Perphenazine OR Piflutixol OR Pimozide OR

Pipamperone OR Prochlorperazine OR Promazine OR Quetiapine OR Raclopride OR Remoxipride OR Reserpine OR Rimcazole OR Risperidone OR Ritanserin OR Sertindole OR Spiperone OR Stepholidine OR Sulforidazine OR Sulpiride OR Sultopride OR Tetrabenazine OR Tetrahydropalmatine OR Thioridazine OR Thiothixene OR "Tiapride Hydrochloride" OR Timiperone OR Trifluoperazine OR Trifluperidol OR Triflupromazine OR Vegetamin OR Veralipride OR Zetidoline OR Ziprasidone OR Zotepine OR Antipsychotic* OR Neuroleptic*).ti,ab.

3.:Cohort Analysis/ OR Case Control Study/ OR Observational Study/ OR Crossover-Procedure/ OR Double-Blind Procedure/ OR Randomized Controlled Trial/ OR Single-Blind Procedure/ OR (Random* OR Factorial* OR Crossover* OR (Cross Over*) OR Cross-Over* OR Placebo* OR (Doubl* adj Blind*) OR (Singl* adj Blind*) OR Assign* OR Allocat* OR Volunteer*).mp. OR (Cohort OR Concurrent OR "Follow Up" OR Followup OR "Incidence Study" OR "Incidence Studies" OR Longitudinal OR Prospective OR Retrospective OR "Case Control" OR Observational OR "Self Controlled" OR "Case Series").af.

4.:1 AND 2 AND 3.

D. MEDLINE

1.:Mortality/ OR Death/ OR Cause of Death/ OR (Mortalit* OR Fatality OR Death).af. OR Mortality.fs.

2.:"Antipsychotic Agents"/ OR Acepromazine/ OR Amoxapine/ OR Aripiprazole/ OR Azaperone/ OR Benperidol/ OR Butaclamol/ OR Chlorpromazine/ OR Chlorprothixene/ OR Clopenthixol/ OR Clozapine/ OR Droperidol/ OR Etazolate/ OR Flupenthixol/ OR Fluphenazine/ OR Fluspirilene/ OR Haloperidol/ OR Loxapine/ OR "Lurasidone Hydrochloride"/ OR Mesoridazine/ OR Methiothepin/ OR Methotrimeprazine/ OR Molindone/ OR Ondansetron/ OR "Paliperidone Palmitate"/ OR Penfluridol/ OR Perazine/ OR Perphenazine/ OR Pimozide/ OR Prochlorperazine/ OR Promazine/ OR "Quetiapine Fumarate"/ OR Raclopride/ OR Remoxipride/ OR Reserpine/ OR Risperidone/ OR Ritanserin/ OR Spiperone/ OR Sulpiride/ OR Tetrabenazine/ OR Thioridazine/ OR Thiothixene/ OR "Tiapride Hydrochloride"/ OR Trifluoperazine/ OR Trifluperidol/ OR Triflupromazine/ OR (Acepromazineh OR Aceprometazine OR Amoxapine OR Amperozide OR Aripiprazole OR Asenapine OR Azaperone OR Benperidol OR Bifeprunox OR Bromperidol OR Butaclamol OR Cariprazine OR Chlorpromazine OR Chlorprothixene OR Clopenthixol OR Clothiapine OR Clozapine OR Dapiprazole OR Dicarbine OR Dihydrohaloperidol OR Dixyrazine OR Droperidol OR Ecopipam OR Etazolate OR Fananserin OR Fencamfamine OR Fluanisone OR Flupenthixol OR Fluperlapine OR Fluphenazine OR Fluspirilene OR Haloperidol OR Iloperidone OR Isofloxythepin OR Lenperone OR Loxapine OR Lurasidone OR Mafoprazine OR Mesoridazine OR Methiothepin OR Methotrimeprazine OR Metylperon OR Molindone OR Nemonapride OR Olanzapine OR Ondansetron OR Oxypertine OR Oxyprothepine OR Paliperidone OR Penfluridol OR Perazine OR Perospirone OR Perphenazine OR Piflutixol OR Pimozide OR Pipamperone OR Prochlorperazine OR Promazine OR Quetiapine OR Raclopride OR Remoxipride OR Reserpine OR Rimcazole OR Risperidone OR Ritanserin OR Sertindole OR Spiperone OR Stepholidine OR Sulforidazine OR Sulpiride OR Sultopride OR Tetrabenazine OR Tetrahydropalmatine OR Thioridazine OR Thiothixene OR "Tiapride Hydrochloride" OR Timiperone OR Trifluoperazine OR Trifluperidol OR Triflupromazine OR Vegetamin OR Veralipride OR Zetidoline OR Ziprasidone OR Zotepine OR Antipsychotic* OR Neuroleptic*).ti,ab.

3.:Cohort Studies/ OR Case-Control Studies/ OR Observational Studies as Topic/ OR (Randomized Controlled Trial OR Controlled Clinical Trial OR Pragmatic Clinical Trial OR Observational Study).pt. OR (Randomi?ed OR Randomly OR Placebo OR Trial OR Groups).ab. OR Drug Therapy.fs. OR (Cohort OR Concurrent OR "Follow Up" OR Followup OR "Incidence Study" OR "Incidence Studies" OR Longitudinal OR Prospective OR Retrospective OR "Case Control" OR Observational OR "Self Controlled" OR "Case Series").af.

4.:1 AND 2 AND 3.

E. PSYCINFO 1806 TO JUNE WEEK 3 2016

1.:Exp "Death and Dying"/ OR (Mortalit* OR Fatality OR Death).af.

2.:Exp Neuroleptic Drugs/ OR (Acepromazineh OR Aceprometazine OR Amoxapine OR Amperozide OR Aripiprazole OR Asenapine OR Azaperone OR Benperidol OR Bifeprunox OR Bromperidol OR Butaclamol OR Cariprazine OR Chlorpromazine OR Chlorprothixene OR Clopenthixol OR Clothiapine OR Clozapine OR Dapiprazole OR Dicarbine OR Dihydrohaloperidol OR Dixyrazine OR Droperidol OR Ecopipam OR Etazolate OR Fananserin OR Fencamfamine OR Fluanisone OR Flupenthixol OR Fluperlapine OR Fluphenazine OR Fluspirilene OR Haloperidol OR Iloperidone OR Isofloxythepin OR Lenperone OR Loxapine OR Lurasidone OR Mafoprazine OR Mesoridazine OR Methiothepin OR Methotrimeprazine OR Metylperon OR Molindone OR Nemonapride OR Olanzapine OR Ondansetron OR Oxypertine OR Oxyprothepine OR Paliperidone OR Penfluridol OR Perazine OR Perospirone OR Perphenazine OR Piflutixol OR Pimozide OR Pipamperone OR Prochlorperazine OR Promazine OR Quetiapine OR Raclopride OR Remoxipride OR Reserpine OR Rimcazole OR Risperidone OR Ritanserin OR Sertindole OR Spiperone OR Stepholidine OR Sulforidazine OR Sulpiride OR Sultopride OR Tetrabenazine OR Tetrahydropalmatine OR Thioridazine

OR Thiothixene OR "Tiapride Hydrochloride" OR Timiperone OR Trifluoperazine OR Trifluperidol OR Triflupromazine OR Vegetamin OR Veralipride OR Zetidoline OR Ziprasidone OR Zotepine OR Antipsychotic* OR Neuroleptic*).ti,ab.

3.:Cohort Analysis/ OR Exp Observation Methods/ OR Exp Treatment Effectiveness Evaluation/ OR Exp Mental Health Program Evaluation/ OR (Randomi$ OR (Random$ Adj (Assign$ OR Allocate$)) OR ((Singl$ OR Doubl$ OR Trebl$ OR Tripl$) adj (Blind$ OR Mask$)) OR Crossover$ OR Cross Over$ OR (Control$ adj2 Trial$)).mp.

4.:1 AND 2 AND 3.

F. PUBMED

("Mortality"[Mesh:NoExp] OR "Death"[Mesh:NoExp] OR "Cause of Death"[Mesh] OR Mortalit*[All Fields] OR Fatality[All Fields] OR Death[All Fields]) AND ("Antipsychotic Agents"[Mesh] OR "Antipsychotic Agents"[Pharmacological Action] OR Acepromazine[Mesh] OR Aceprometazine[Supplementary Concept] OR Amoxapine[Mesh] OR Amperozide[Supplementary Concept] OR "Aripiprazole Lauroxil"[Supplementary Concept] OR Aripiprazole[Mesh] OR Asenapine[Supplementary Concept] OR Azaperone[Mesh] OR Benperidol[Mesh] OR Bifeprunox[Supplementary Concept] OR "Bromperidol Decanoate"[Supplementary Concept] OR Bromperidol[Supplementary Concept] OR Butaclamol[Mesh] OR Cariprazine[Supplementary Concept] OR Chlorpromazine[Mesh] OR Chlorprothixene[Mesh] OR "Clopenthixol Acetate Ester"[Supplementary Concept] OR "Clopenthixol Decanoate"[Supplementary Concept] OR Clopenthixol[Mesh] OR Clothiapine[Supplementary Concept] OR Clozapine[Mesh] OR Dapiprazole[Supplementary Concept] OR Dicarbine[Supplementary Concept] OR Dihydrohaloperidol[Supplementary Concept] OR Dixyrazine[Supplementary Concept] OR Droperidol[Mesh] OR Ecopipam[Supplementary Concept] OR Etazolate[Mesh] OR Fananserin[Supplementary Concept] OR Fencamfamine[Supplementary Concept] OR Fluanisone[Supplementary Concept] OR Flupenthixol[Mesh] OR Fluperlapine[Supplementary Concept] OR "Fluphenazine Depot"[Supplementary Concept] OR "Fluphenazine Enanthate"[Supplementary Concept] OR Fluphenazine[Mesh] OR Fluspirilene[Mesh] OR "Haloperidol Decanoate"[Supplementary Concept] OR Haloperidol[Mesh] OR Iloperidone[Supplementary Concept] OR Isofloxythepin[Supplementary Concept] OR Lenperone[Supplementary Concept] OR Loxapine[Mesh] OR "Lurasidone Hydrochloride"[Mesh] OR Mafoprazine[Supplementary Concept] OR Mesoridazine[Mesh] OR Methiothepin[Mesh] OR Methotrimeprazine[Mesh] OR Metylperon[Supplementary Concept] OR Molindone[Mesh] OR Nemonapride[Supplementary Concept] OR Olanzapine[Supplementary Concept] OR Ondansetron[Mesh] OR Oxypertine[Supplementary Concept] OR "Paliperidone Palmitate"[Mesh] OR Penfluridol[Mesh] OR Perazine[Mesh] OR Perospirone[Supplementary Concept] OR Perphenazine[Mesh] OR Piflutixol[Supplementary Concept] OR Pimozide[Mesh] OR Pipamperone[Supplementary Concept] OR Prochlorperazine[Mesh] OR Promazine[Mesh] OR "Quetiapine Fumarate"[Mesh] OR Raclopride[Mesh] OR Remoxipride[Mesh] OR Reserpine[Mesh] OR Rimcazole[Supplementary Concept] OR Risperidone[Mesh] OR Ritanserin[Mesh] OR Sertindole[Supplementary Concept] OR Spiperone[Mesh] OR Stepholidine[Supplementary Concept] OR Sulforidazine[Supplementary Concept] OR Sulpiride[Mesh] OR Sultopride[Supplementary Concept] OR Tetrabenazine[Mesh] OR Tetrahydropalmatine[Supplementary Concept] OR Thioridazine[Mesh] OR Thiothixene[Mesh] OR "Tiapride Hydrochloride"[Mesh] OR Timiperone[Supplementary Concept] OR Trifluoperazine[Mesh] OR Trifluperidol[Mesh] OR Triflupromazine[Mesh] OR Vegetamin[Supplementary Concept] OR Veralipride[Supplementary Concept] OR Zetidoline[Supplementary Concept] OR Ziprasidone[Supplementary Concept] OR Zotepine[Supplementary Concept] OR Aceprometazine[tiab] OR Amoxapine[tiab] OR Amperozide[tiab] OR Aripiprazole[tiab] OR Asenapine[tiab] OR Azaperone[tiab] OR Benperidol[tiab] OR Bifeprunox[tiab] OR Bromperidol[tiab] OR Butaclamol[tiab] OR Cariprazine[tiab] OR Chlorpromazine[tiab] OR Chlorprothixene[tiab] OR Clopenthixol[tiab] OR Clothiapine[tiab] OR Clozapine[tiab] OR Dapiprazole[tiab] OR Dihydrohaloperidol[tiab] OR Dixyrazine[tiab] OR Droperidol[tiab] OR Ecopipam[tiab] OR Etazolate[tiab] OR Fananserin[tiab] OR Fencamfamine[tiab] OR Fluanisone[tiab] OR Flupenthixol[tiab] OR Fluperlapine[tiab] OR Fluphenazine[tiab] OR Fluspirilene[tiab] OR Haloperidol[tiab] OR Iloperidone[tiab] OR Isofloxythepin[tiab] OR Lenperone[tiab] OR Loxapine[tiab] OR Lurasidone[tiab] OR Mafoprazine[tiab] OR Mesoridazine[tiab] OR Methiothepin[tiab] OR Methotrimeprazine[tiab] OR Metylperon[tiab] OR Molindone[tiab] OR Nemonapride[tiab] OR Olanzapine[tiab] OR Ondansetron[tiab] OR Oxypertine[tiab] OR Oxyprothepine[tiab] OR Paliperidone[tiab] OR Penfluridol[tiab] OR Perazine[tiab] OR Perospirone[tiab] OR Perphenazine[tiab] OR Piflutixol[tiab] OR Pimozide[tiab] OR Pipamperone[tiab] OR Prochlorperazine[tiab] OR Promazine[tiab] OR Quetiapine[tiab] OR Raclopride[tiab] OR Remoxipride[tiab] OR Reserpine[tiab] OR Rimcazole[tiab] OR Risperidone[tiab] OR Ritanserin[tiab] OR Sertindole[tiab] OR Spiperone[tiab] OR Stepholidine[tiab] OR Sulforidazine[tiab] OR Sulpiride[tiab] OR Sultopride[tiab] OR Tetrabenazine[tiab] OR Tetrahydropalmatine[tiab] OR Thioridazine[tiab] OR Thiothixene[tiab] OR "Tiapride Hydrochloride"[tiab] OR Timiperone[tiab] OR Trifluoperazine[tiab] OR Trifluperidol[tiab] OR Triflupromazine[tiab] OR Vegetamin[tiab] OR Veralipride[tiab] OR Zetidoline[tiab] OR

Ziprasidone[tiab] OR Zotepine[tiab] OR Antipsychotic*[tiab] OR Neuroleptic*[tiab]) AND (Randomized Controlled Trial[pt] OR Controlled Clinical Trial[pt] OR Pragmatic Clinical Trial[pt] OR Randomized[tiab] OR Randomised[tiab] OR Placebo[tiab] OR Randomly[tiab] OR Trial[tiab] OR Groups[tiab] OR "Cohort Studies"[Mesh] OR "Case-Control Studies"[Mesh] OR "Observational Study"[pt] OR "Observational Studies as Topic"[Mesh] OR Cohort[All Fields] OR Concurrent[All Fields] OR "Follow Up"[All Fields] OR Followup[All Fields] OR "Incidence Study"[All Fields] OR "Incidence Studies"[All Fields] OR Longitudinal[All Fields] OR Prospective[All Fields] OR Retrospective[All Fields] OR "Case Control"[All Fields] OR Observational[All Fields] OR "Self Controlled"[All Fields] OR "Case Series"[All Fields]) NOT MEDLINE[sb].

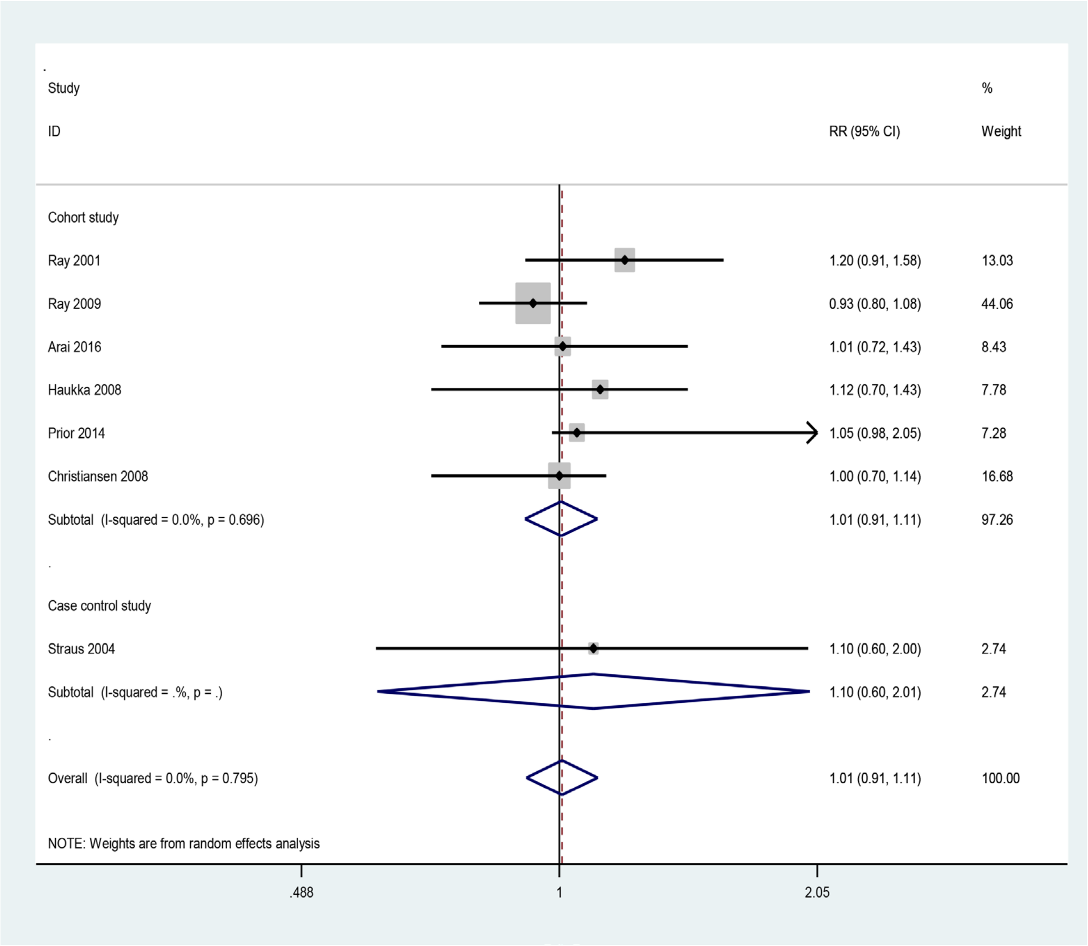

Supplementary Figure 1: All cause mortality of past AP users vs. AP non-users.

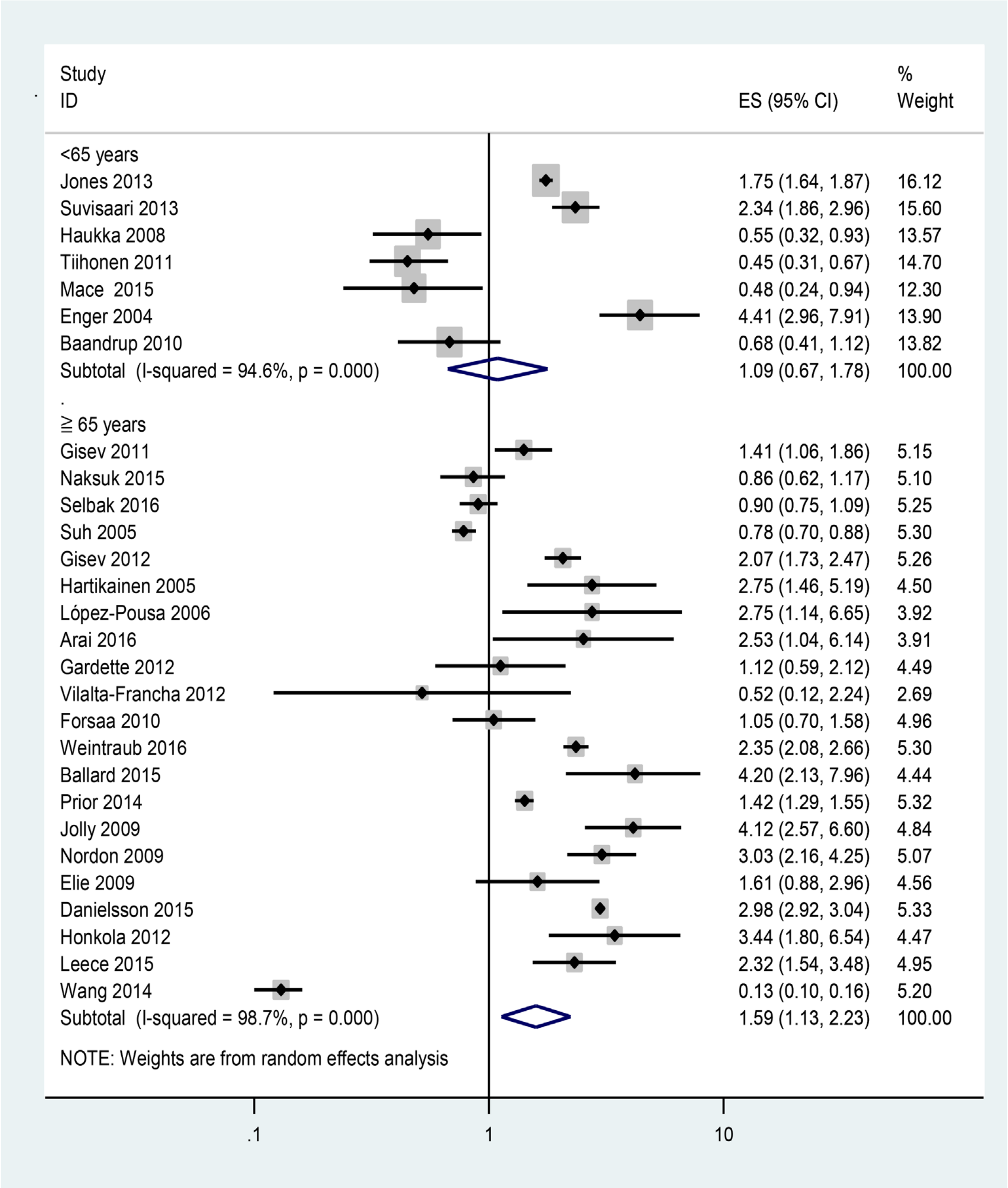

Supplementary Figure 2: All cause mortality of current AP user vs AP non-users for different age.

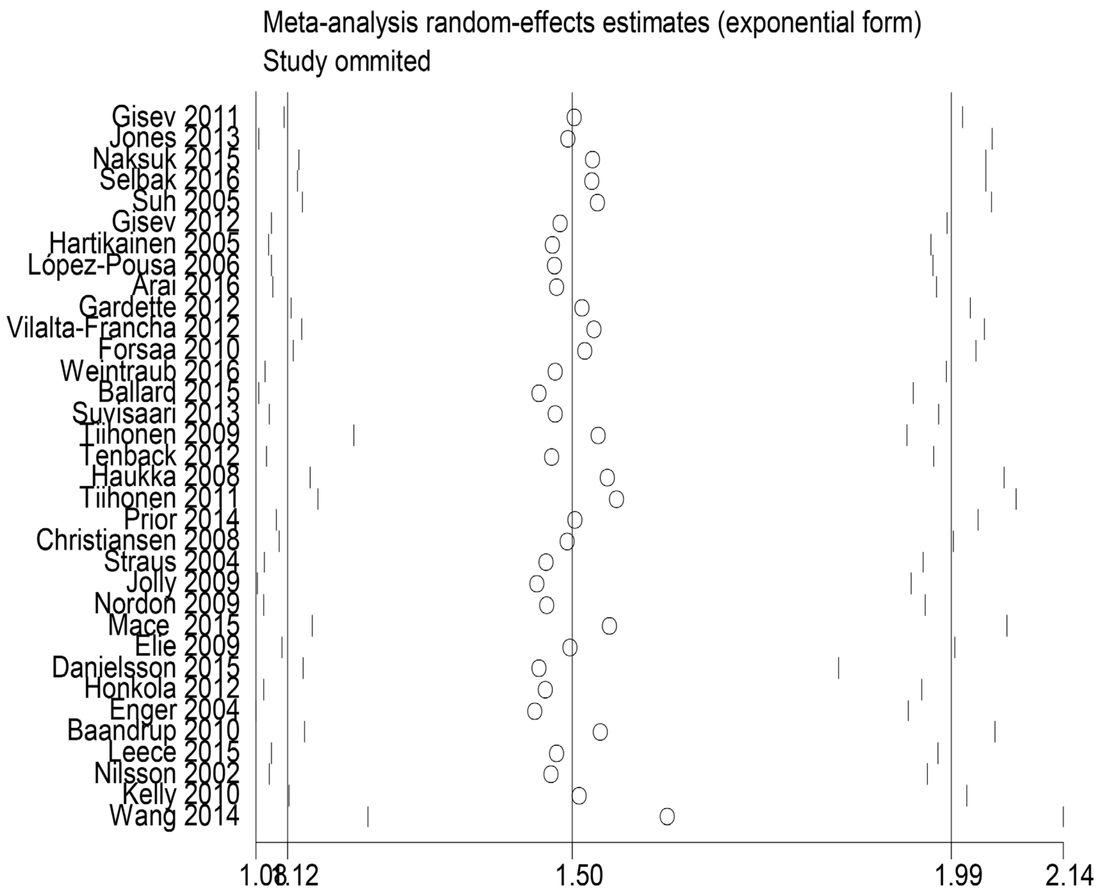

Supplementary Figure 3: Sensitivity analyses of a single study in meta-analysis estimation.

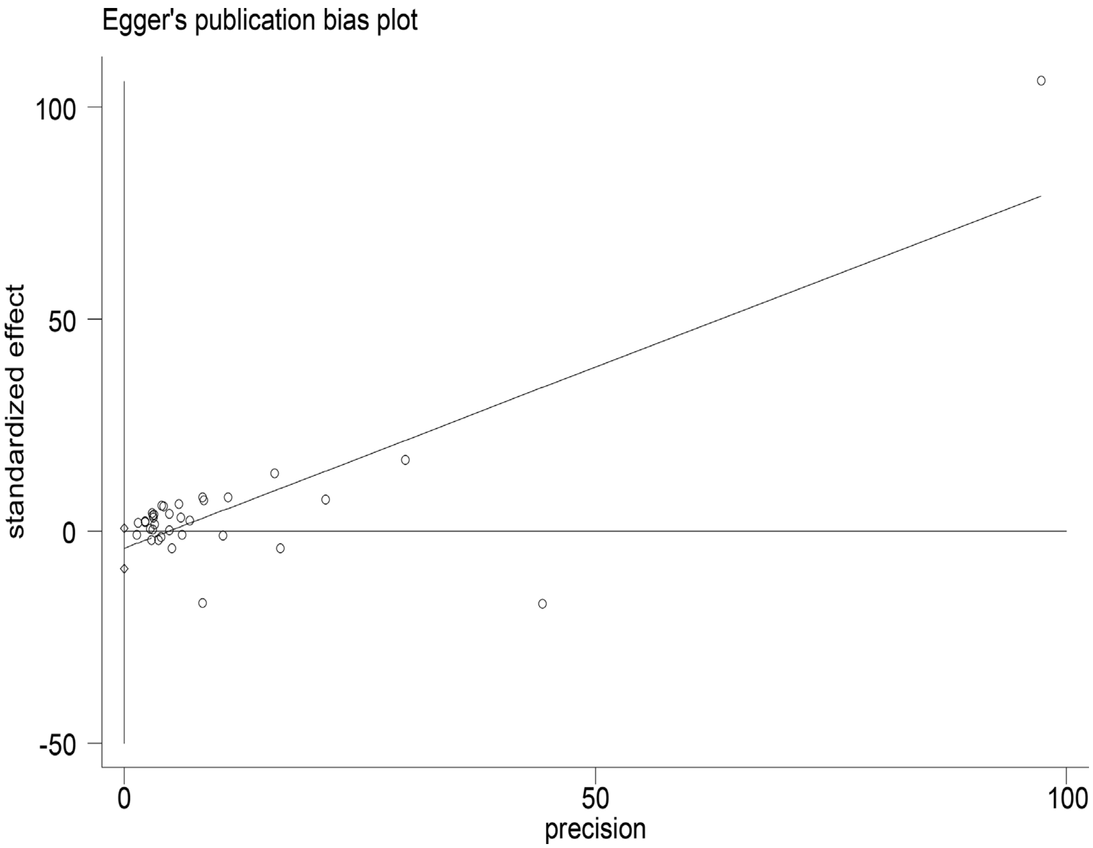

Supplementary Figure 4: Funnel plot of all cause mortality for AP users vs AP non-users.

Supplementary Table 1: The characteristic of included studies. See_Supplementary_Table 1

Supplementary Table 1: Risk of bias in included studies. See_Supplementary_Table 2

1. Straus SM, Bleumink GS, Dieleman JP, van der Lei J, 't Jong GW, Kingma JH, Sturkenboom MC, Stricker BH. Antipsychotics and the risk of sudden cardiac death. Archives of internal medicine. 2004; 164:1293–1297.
2. Ray W A, Meredith S, Thapa PB, Meador KG, Hall K, Murray KT. Antipsychotics and the risk of sudden cardiac death. Archives of General Psychiatry. 2001; 58:1161–1167.
3. Ray WA, Chung CP, Murray KT, Hall K, Stein CM. Atypical antipsychotic drugs and the risk of sudden cardiac death. New England Journal of Medicine. 2009; 360:225–235.
4. Gisev N, Hartikainen S, Chen TF, Korhonen, M, Bell JS. Mortality associated with benzodiazepines and benzodiazepine-related drugs among community-dwelling older people in Finland: a population-based retrospective cohort study. The Canadian Journal of Psychiatry. 2011; 56:377–381.
5. Aparasu RR, Chatterjee S, Mehta S, Chen H. Risk of death in dual-eligible nursing home residents using typical or atypical antipsychotic agents. Medical care. 2012; 50:961–969.
6. Wang PS, Schneeweiss S, Avorn J, Fischer MA, Mogun H, Solomon DH, Brookhart MA. Risk of death in elderly users of conventional vs. atypical antipsychotic medications. New England Journal of Medicine. 2005; 353:2335–2341.
7. Murray-Thomas T, Jones ME, Patel D, Brunner E, Shatapathy CC, Motsko S, Van Staa TP. Risk of Mortality (Including Sudden Cardiac Death) and Major Cardiovascular Events in Atypical and Typical Antipsychotic Users: A Study with the General Practice Research Database. 2013; 2013:647476.
8. Jolly K, Gammage MD, Cheng KK, Bradburn P, Banting, MV, Langman MJ. Sudden death in patients receiving drugs tending to prolong the QT interval. British journal of clinical pharmacology. 2009; 68:743–751.
9. Sikirica S, Marino M, Gagne JJ, Palma R, Maio V. Risk of death associated with the use of conventional vs. atypical antipsychotic medications: evaluating the use of the Emilia-Romagna Region database for pharmacoepidemiological studies. Journal of clinical pharmacy and therapeutics. 2014; 39:38–44.
10. Nordon C, Martin-Latry K, de Roquefeuil L, Latry P, Bégaud B, Falissard B, Rouillon F, Verdoux H. Risk of death related to psychotropic drug use in older people during the European 2003 heat wave: a population-based case–control study. The American Journal of Geriatric Psychiatry. 2009; 17:1059–1067.
11. Hwang YJ, Dixon SN, Reiss JP, Wald R, Parikh CR, Gandhi S, Shariff SZ, Pannu N, Nash DM, Rehman F, Garg AX. Atypical antipsychotic drugs and the risk for acute kidney injury and other adverse outcomes in older adults: a population-based cohort study. Annals of internal medicine. 2014; 161:242–248.
12. Mace S, Dzahini O, Cornelius V, Anthony D, Stewart R, Taylor D. Antipsychotic use and unexpected death: a hospital-based case–control study. Acta Psychiatrica Scandinavica. 2015; 132:479–488.
13. Park Y, Franklin JM, Schneeweiss S, Levin R, Crystal S, Gerhard T, Huybrechts KF. Antipsychotics and mortality: adjusting for mortality risk scores to address confounding by terminal illness. Journal of the American Geriatrics Society. 2015; 63:516–523.
14. Setoguchi S, Wang P S, Alan Brookhart M, Canning C F, Kaci L, Schneeweiss S. Potential causes of higher mortality in elderly users of conventional and atypical antipsychotic medications. Journal of the American Geriatrics Society. 2008; 56:1644–1650.
15. Schneeweiss S, Setoguchi S, Brookhart A, Dormuth C, Wang PS. Risk of death associated with the use of conventional versus atypical antipsychotic drugs among elderly patients. Canadian Medical Association Journal. 2007; 176:627–632.
16. Pratt N, Roughead EE, Ryan P, Salter A. Antipsychotics and the risk of death in the elderly: an instrumental variable analysis using two preference based instruments. Pharmacoepidemiology and drug safety. 2010; 19:699–707.
17. Elie M, Boss K, Cole MG, McCusker J, Belzile E, Ciampi A. A retrospective, exploratory, secondary analysis of the association between antipsychotic use and mortality in elderly patients with delirium. International Psychogeriatrics. 2009; 21:588–592.
18. Naksuk N, Thongprayoon C, Park JY, Sharma S, Gaba P, Rosenbaum AN, Peeraphatdit T, Hu TY, Bell MR, Herasevich V, Brady PA, Kapa S, Asirvatham SJ. Clinical impact of delirium and antipsychotic therapy: 10-Year experience from a referral coronary care unit. European heart journal. Acute cardiovascular care. 2017; 6:560-568.
19. Selbæk G, Aarsland D, Ballard C, Engedal K, Langballe EM, Benth JŠ, Bergh S. Antipsychotic Drug Use Is Not Associated With Long-Term Mortality Risk in Norwegian Nursing Home Patients. Journal of the American Medical Directors Association. 2016; 17:464.e1–7.
20. Huybrechts KF, Rothman KJ, Silliman RA, Brookhart MA, Schneeweiss S. Risk of death and hospital admission for major medical events after initiation of psychotropic medications in older adults admitted to nursing homes. Canadian Medical Association Journal. 2011; 183:e411–e419.
21. Nonino F, De Girolamo G, Gamberini L, Goldoni C A, Modena Work Group for Antipsychotics in Dementia. Survival among elderly Italian patients with dementia treated with atypical antipsychotics: observational study. Neurological Sciences. 2006; 27:375–380.
22. Trifrò G, Verhamme K, Ziere G, Caputi A P, Ch Stricker B H, Sturkenboom M C. All-cause mortality associated with atypical and typical antipsychotics in demented outpatients. Pharmacoepidemiology and drug safety. 2007; 16:538–544.
23. Simoni-Wastila L, Ryder PT, Qian J, Zuckerman IH, Shaffer T, Zhao L. Association of antipsychotic use with hospital events and mortality among medicare beneficiaries residing in long-term care facilities. The American Journal of Geriatric Psychiatry. 2009; 17:417–427.
24. Huybrechts KF, Brookhart MA, Rothman KJ, Silliman RA, Gerhard T, Crystal S, Schneeweiss S. Comparison of different approaches to confounding adjustment in a study on the association of antipsychotic medication with mortality in older nursing home patients. American journal of epidemiology. 2011; 174:1089–1099.
25. Suh GH, Shah A. Effect of antipsychotics on mortality in elderly patients with dementia: a 1-year prospective study in a nursing home. International Psychogeriatrics. 2005; 17:429–441.
26. Gisev N, Hartikainen S, Chen TF, Korhonen M, Bell JS. Effect of comorbidity on the risk of death associated with antipsychotic use among community-dwelling older adults. International Psychogeriatrics. 2012; 24:1058–1064.
27. Kales HC, Valenstein M, Kim HM, McCarthy JF, Ganoczy D, Cunningham F, Blow FC. Mortality risk in patients with dementia treated with antipsychotics versus other psychiatric medications. American Journal of Psychiatry. 2007; 164:1568–76.

28. Hartikainen S, Rahkonen T, Kautiainen H, Sulkava R. The use of psychotropics and survival in demented elderly individuals. International clinical psychopharmacology. 2005; 20:227–231.

29. Liperoti R, Onder G, Landi F, Lapane KL, Mor V, Bernabei R, Gambassi G. All-cause mortality associated with atypical and conventional antipsychotics among nursing home residents with dementia: a retrospective cohort study. The Journal of clinical psychiatry. 2009; 70:1340–1347.

30. Gill SS, Bronskill SE, Normand SL, Anderson GM, Sykora K, Lam K, Bell CM, Lee PE, Fischer HD, Herrmann N, Gurwitz JH, Rochon PA. Antipsychotic drug use and mortality in older adults with dementia. Annals of internal medicine. 2007; 146:775–786.

31. Piersanti M, Capannolo M, Turchetti M, Serroni N, De Berardis D, Evangelista P, Costantini P, Orsini A, Rossi A, Maggio R. Increase in mortality rate in patients with dementia treated with atypical antipsychotics: a cohort study in outpatients in Central Italy. Riv Psichiatr. 2014; 49:34–40.

32. Raivio MM, Laurila JV, Strandberg TE, Tilvis R S, Pitkälä KH. Neither atypical nor conventional antipsychotics increase mortality or hospital admissions among elderly patients with dementia: a two-year prospective study. The American journal of geriatric psychiatry. 2007; 15:416–424.

33. Connors MH, Ames D, Boundy K, Clarnette R, Kurrle S, Mander A, Ward J, Woodward M, Brodaty H. Predictors of Mortality in Dementia: The PRIME Study. Journal of Alzheimer's disease. 2016; 52:967–974.

34. Jackson JW, VanderWeele TJ, Viswanathan A, Blacker D, Schneeweiss S. The explanatory role of stroke as a mediator of the mortality risk difference between older adults who initiate first-versus second-generation antipsychotic drugs. American journal of epidemiology. 2014; 180:847–852.

35. Sultana J, Chang CK, Hayes RD, Broadbent M, Stewart R, Corbett A, Ballard C. Associations between risk of mortality and atypical antipsychotic use in vascular dementia: a clinical cohort study. International journal of geriatric psychiatry. 2014; 29:1249–1254.

36. Musicco M, Palmer K, Russo A, Caltagirone C, Adorni F, Pettenati C, Bisanti L. Association between prescription of conventional or atypical antipsychotic drugs and mortality in older persons with Alzheimer's disease. Dementia and geriatric cognitive disorders. 2011; 31:218–224.

37. López-Pousa S, Olmo JG, Franch JV, Estrada AT, Cors OS, Nierga IP, Gelada-Batlle E. Comparative analysis of mortality in patients with Alzheimer's disease treated with donepezil or galantamine. Age and ageing. 2006; 35:365–371.

38. Arai H, Nakamura Y, Taguchi M, Kobayashi H, Yamauchi K, Schneider LS and J-CATIA Study Group. Mortality risk in current and new antipsychotic AD users: Large scale Japanese study. Alzheimer›s & Dementia. 2016; 12:823–30.

39. Lopez OL, Becker JT, Chang YF, Sweet RA, Aizenstein H, Snitz B, Saxton J, McDade E, Kamboh MI, DeKosky ST, Reynolds CF 3rd, Klunk WE. The Long-Term Effects of Conventional and Atypical Antipsychotics in Patients With Probable Alzheimer's Disease. Am J Psychiatry. 2013; 170:1051–1058.

40. Gardette V, Lapeyre-Mestre M, Coley N, Cantet C, Montastruc JL, Vellas B, Andrieu S. Antipsychotic use and mortality risk in community-dwelling Alzheimer's disease patients: evidence for a role of dementia severity. Current Alzheimer Research. 2012; 9:1106–1116.

41. Vilalta-Franch J, Calvó-Perxas L, Garre-Olmo J, Turró-Garriga O, López-Pousa S. Apathy syndrome in Alzheimer›s disease epidemiology: prevalence, incidence, persistence, and risk and mortality factors. Journal of Alzheimer's Disease. 2013; 33:535–543.

42. Danielsson B, Collin J, Jonasdottir B G, Borg N, Salmi P, Fastbom J. Antidepressants and antipsychotics classified with torsades de pointes arrhythmia risk and mortality in older adults-a Swedish nationwide study. British journal of clinical pharmacology. 2016; 81:773–783.

43. Frandsen R, Baandrup L, Kjellberg J, Ibsen R, Jennum P. Increased all-cause mortality with psychotropic medication in Parkinson›s disease and controls: A national register-based study. Parkinsonism & related disorders. 2014; 20:1124–1128.

44. Forsaa E B, Larsen JP, Wentzel-Larsen T, Alves G. What predicts mortality in Parkinson disease? A prospective population-based long-term study. Neurology. 2010; 75:1270–1276.

45. Weintraub D, Chiang C, Kim HM, Wilkinson J, Marras C, Stanislawski B, Mamikonyan E, Kales HC. Association of antipsychotic use with mortality risk in patients with Parkinson disease. JAMA neurology. 2016; 73:535–541.

46. Ballard C, Isaacson S, Mills R, Williams H, Corbett A, Coate B, Pahwa R, Rascol O, Burn DJ. Impact of current antipsychotic medications on comparative mortality and adverse events in people with Parkinson disease psychosis. Journal of the American Medical Directors Association. 2015; 16:898.e1–7.

47. Marras C, Gruneir A, Wang X, Fischer H, Gill SS, Herrmann N, Anderson GM, Hyson C, Rochon PA. Antipsychotics and mortality in Parkinsonism. The American Journal of Geriatric Psychiatry. 2012; 20:149–158.

48. Jackson J W, Vander Weele T J, Blacker D, Schneeweiss S. Mediators of first-versus second-generation antipsychotic related mortality in older adults. Epidemiology (Cambridge, Mass.). 2015; 26:700.

49. Suvisaari J, Partti K, Perälä J, Viertiö S, Saarni SE, Lönnqvist J, Saarni SI, Härkänen T. Mortality and its determinants in people with psychotic disorder. Psychosomatic medicine. 2013; 75:60–67.

50. Honkola J1, Hookana E, Malinen S, Kaikkonen KS, Junttila MJ, Isohanni M, Kortelainen ML, Huikuri HV. Psychotropic medications and the risk of sudden cardiac death during an acute coronary event. European heart journal. 2012; 33:745–751.

51. Kelly DL, McMahon RP, Liu F, Love RC, Wehring HJ, Shim JC, Warren KR, Conley RR. Cardiovascular disease mortality in patients with chronic schizophrenia treated with clozapine: a retrospective cohort study. The Journal of clinical psychiatry. 2010; 71:304–311.

52. Tiihonen J, Lönnqvist J, Wahlbeck K, Klaukka T, Niskanen L, Tanskanen A, Haukka J. 11-year follow-up of mortality in patients with schizophrenia: a population-based cohort study (FIN11study). Lancet. 2009; 374:620–7.

53. Tenback D, Pijl B, Smeets H, van Os J, van Harten P. All-cause mortality and medication risk factors in schizophrenia: a prospective cohort study. Journal of clinical psychopharmacology. 2012; 32:31–35.

54. Kiviniemi M, Suvisaari J, Koivumaa-Honkanen H, Häkkinen U, Isohanni M, Hakko H. Antipsychotics and mortality in first-onset schizophrenia: prospective Finnish register study with 5-year follow-up. Schizophrenia research. 2013; 150:274–280.

55. Haukka J, Tiihonen J, Härkänen T, Lönnqvist J. Association between medication and risk of suicide, attempted suicide and death in nationwide cohort of suicidal patients with schizophrenia. Pharmacoepidemiology and drug safety. 2008; 17:686–696.
56. Enger C, Weatherby L, Reynolds RF, Glasser D B, Walker AM. Serious cardiovascular events and mortality among patients with schizophrenia. The Journal of nervous and mental disease. 2004; 192:19–27.
57. Baandrup L, Gasse C, Jensen VD, Glenthoj BY, Nordentoft M, Lublin H, Fink-Jensen A, Lindhardt A, Mortensen PB. Antipsychotic polypharmacy and risk of death from natural causes in patients with schizophrenia: a population-based nested case-control study. The Journal of clinical psychiatry. 2009; 71:103–108.
58. Hou PY, Hung GCL, Jhong JR, Tsai SY, Chen CC, Kuo CJ. Risk factors for sudden cardiac death among patients with schizophrenia. Schizophrenia research. 2015; 168:395–401.
59. Chen VC, Liao YT, Lai TJ, Lane HY, Shao WC, Dewey M, Lee CT, Lu ML. Survival analysis of the use of first and second generation antipsychotics among patients suffering schizophrenia: A nationwide population-based cohort study. Schizophrenia research. 2015; 169:406–411.
60. Tiihonen J, Haukka J, Taylor M, Haddad PM, Patel MX, Korhonen P. A nationwide cohort study of oral and depot antipsychotics after first hospitalization for schizophrenia. American Journal of Psychiatry. 2011; 168:603–609.
61. Murray-Thomas T, Jones ME, Patel D, Brunner E, Shatapathy CC, Motsko S, Van Staa TP. Risk of mortality (including sudden cardiac death) and major cardiovascular events in atypical and typical antipsychotic users: a study with the general practice research database. Cardiovascular psychiatry and neurology. 2013; 2013:247486.
62. Prior A, Laursen TM, Larsen KK, Johnsen SP, Christensen J, Andersen G, Vestergaard M. Post-stroke mortality, stroke severity, and preadmission antipsychotic medicine use–A population based cohort study. PLoS one. 2014; 9:e84103.
63. Wang JY, Wang CY, Tan CH, Chao TT, Huang YS, Lee CC. Effect of different antipsychotic drugs on short-term mortality in stroke patients. Medicine. 2014; 93.
64. Leece P, Cavacuiti C, Macdonald EM, Gomes T, Kahan M, Srivastava A, Steele L, Luo J, Mamdani MM, Juurlink DN and Canadian Drug Safety and Effectiveness Research Network. Predictors of opioid-related death during methadone therapy. Journal of substance abuse treatment. 2015; 57:30–35.
65. Acharya T, Acharya S, Tringali S, Huang J. Association of antidepressant and atypical antipsychotic use with cardiovascular events and mortality in a veteran population. Pharmacotherapy: The Journal of Human Pharmacology and Drug Therapy. 2013; 33:1053–1061.
66. Nilsson L, Ahlbom A, Farahmand BY, Åsberg M, Tomson T. Risk factors for suicide in epilepsy: a case control study. Epilepsia. 2002; 43:644–651.
67. Christiansen C, Christensen S, Riis A, Thomsen RW, Johnsen SP, Tonnesen E, Sorensen HT. Antipsychotic drugs and short-term mortality after peptic ulcer perforation: a population-based cohort study. Alimentary pharmacology & therapeutics. 2008; 28:895–902.
68. Barnett MJ, Perry PJ, Alexander B, Kaboli, PJ. Risk of mortality associated with antipsychotic and other neuropsychiatric drugs in pneumonia patients. Journal of clinical psychopharmacology. 2006; 26:182–185.
