# Supplementary material for: Does antipsychotic drug use increase the risk of long term mortality? A systematic review and meta-analysis of observational studies
Source: Oncotarget. 2018 Jan 10;9(19):15101–10. doi: 10.18632/oncotarget.24120 (PMC5871101; doi:10.18632/oncotarget.24120)
Supplement: Supplementary file 2 [file oncotarget-09-15101-s002.docx]

**Supplementary table 1: The characteristic of included studies**

| **Reference** | **Country** | **Design** | **Setting** | **Study period** | **Disease** | **Age** | **Sample** | **Outcome**  **(follow up period)** | **Confounders measured** | **Results** |
| --- | --- | --- | --- | --- | --- | --- | --- | --- | --- | --- |
| Straus 2004 [1] | Netherlands | case-control study | longitudinal observational database | 1995-2001 | AP users | ≧18 years | 5017 | sudden cardiac death | age, sex, practice, calendar time, disease, use of diuretics | 1.AP vs none:  OR=3.3 (1.8-6.2)  2.Past AP vs none:  OR= 1.1 (0.6-2.0) |
| Ray 2001 [2] | US | retrospective cohort study | community setting | 1988-1993 | AP users | 15-84 years | 481744 | sudden cardiac death | calendar year, age, sex, race, noncardiovascular illness, and cardiovascular disease | Past AP vs none  RR=1.20(0.91-1.58); |
| Ray 2009 [3] | US | retrospective cohort study | database | 1990-2003 | AP users | 30-74 years  (mean: 45.7- 46.4 years) | 183893 | sudden cardiac death  (median: 2.2-2.9 years) | age, sex, race, medicaid enrollment due to disability, cardiovascular risk, psychiatric characteristics (propensity score) | 1.CAP vs none  RR=1.84 (1.50-2.26)  2.AAP vs none:  RR=1.99 (1.61-2.46)  3.Past AP vs none:  RR=0.93 (0.80-1.08) |
| Gisev 2011 [4] | Finland | population-based retrospective cohort study | community-dwelling older people | 2000-2008 | AP users | ≧65 years  (mean:73.8-76.1 years) | 1845 | all-cause mortality  (9 years) | age, sex, antipsyehotic drug use, and diagnose | AP vs none:  HR=1.41( 1.06-1.86) |
| Rajender 2012 [5] | US | retrospective cohort study | nursing home residents | 2001–2003 | AP users | ≧65 years  (mean:  ≧80 years) | 7,218 | all-cause mortality (180-day) | age, sex,race, hospitalization, medical history, psychotic disorder, other drugs used | CAP vs AAP HR=1.41( 1.27-1.57) |
| Wang 2005 [6] | US | retrospective cohort study | hospitalizations, nursing home stays, and outpatients | 1994-2003 | AP users | ≧65 years  (mean: 83.2-83.5 years) | 22,890 | all-cause mortality (180-day) | calendar year, age, sex, race, disease, medications, total number of medications used, hospitalizations, and nursing home stays. | CAP vs AAP  HR=1.37(1.27-1.49); |
| Murray-Thomas 2013 [7] | UK | retrospective  cohort study | general practice research database | 1995-2011 | AP users | ≧18 years  (mean: 49-61 years) | 377312 | 1.all-cause mortality 2.cardiac death  (mean:  3.6-4.1 years) | age, sex, socioeconomic status, smoking history, alcohol use, and body mass index (BMI). | **All-cause mortality:**  1.AP vs none:  RR=1.75 (1.64-1.87);  2.CAP vs none:  RR=1.82 (1.69-1.98)  3.AAP vs none:  RR=1.52 (1.40-1.64)  **Cardiac death:**  1.AP vs none:  RR=1.72, (1.42-2.07) 2.CAP vs none:  RR=1.88 (1.49-2.39)  3.AAP vs none:  RR=1.74 (1.40-2.14) |
| Jolly 2009 [8] | UK | case–control study | community | 2003-2007 | AP users | mean 67.6 years | 4040 | 1.cardiac death  2.sudden death | disease, history of alcohol abuse | **Cardiac death**  1.AP vs none:  OR= 6.15 (3.02-12.57)  **Sudden death** 1.AP vs none:  OR=4.12(2.57-6.60)  2.CAP vs none:  OR=3.14(1.53- 6.43)3.AAP vs none:  OR=4.79 (2.67-8.59) |
| Sikirica 2014 [9] | Italy | retrospective cohort study | region database | 2009-2011 | AP users | ≧65 years  (mean: 78.9-82.1 years) | 23681 | all-cause mortality (180 days) | age, sex, home care visits, hospital admissions, medications, combined comorbidity score, disease | CAP vs AAP  HR=1.47 (1.35–1.60) |
| Nordon 2009 [10] | France | retrospective population-based case–control study | social security insurance national database | 2003 | AP users | 70-100 years | 19062 | all-cause mortality | age, gender, and presence of chronic illness | AP vs none  OR=3.03(2.16–4.25) |
| Hwang 2014 [11] | Canada | population-based retrospective cohort study | outpatient | 2003-2012 | AP users | ≧65 years  (mean: 80.7 years) | 195554 | all-cause mortality (90 days) | regional physicians’ offices, hospitals, community mental health and addiction centers, community health centers, and long-term care facilities | AAP vs none  RR=2.39 (2.28–2.50) |
| Mace 2015 [12] | UK | case–control study | hospital-based patients | 2009-2011 | AP users | 18-65 years  (mean: 39-49 years) | 536 | all-cause mortality | age, gender, cardiovascular disease, diagnosis psychotic, opiate use and physical comorbidity | 1.AP vs none  OR=0.48 (0.24–0.94)  2.CAP vs none  OR=0.83 (0.31–2.20)  3.AAP vs none  OR=0.42 (0.21–0.86) |
| Park 2015 [13] | US | prospective cohort study | nursing home residents | 2001-2005 | AP users | ≧65 years | 75445 | all-cause mortality  (within 180 days) | Age, sex, race, education, geographical region, morbidity, nursing home facility | CAP vs AAP  HR=2.20(2.02–2.39) |
| Setoguchi 2008 [14] | UK | cohort study | community | 1996-2004 | AP users | ≧65 years  (mean: 79.88-80.3 years) | 37,241 | all-cause mortality  (180 days) | calendar year, age, sex, race, medications, hospitalization, and nursing home stays, morbidity | **All-cause mortality**  CAP vs AAP  HR=1.27 (1.18–1.37)  **Cardiac death**  CAP vs AAP  HR=1.23 (1.10–1.36) |
| Schneeweiss 2007 [15] | Canada | population-based retrospective cohort study | health care utilization data | 1996-2004 | AP users | ≧65 years  (mean: 79.9-80.3 years) | 37241 | all-cause mortality (180-day) | calendar year, age, sex, race, morbidity, medications, hospital admissions and nursing home stays. | 1.CAP vs AAP  HR=1.11 (1.04–1.19)  2.CAP vs none  HR=1.32 (1.23–1.42) |
| Pratt 2010 [16] | Australia | cohort study | administrative claims database | 2003-2006 | AP users | 80-87 years  (mean: 83 years) | 16,539 | all-cause mortality | age, male, et al (instrumental variables) | CAP vs AAP:  OR=3.5 (3.2–3.8); |
| Elie 2009 [17] | Canada | nested case-control | hospitalized patients | 1996-1999 | delirium | ≧65 years | 2435 | in-hospital mortality | charlson comorbidity Index score, acute physiology score, living arrangement, MMSE score, delirium Index score | AP vs none  OR=1.61(0.88–2.96) |
| Naksuk 2015 [18] | US | cohort study | coronary care unit patients | 2004-2013 | delirium | 67±15 years | 925 | all-cause mortality  (1 year) | age, sex, disease and medications | AP vs none  OR=0.67 (0.42–1.04)删  OR=0.86 (0.62–1.17) |
| Selbæk 2016 [19] | Norway | prospective cohort study | nursing home patients | 2004-2005 | AP users | mean  84.4 years | 1163 | all-cause mortality  (75 months) | age, male, education, marital status, disease, days in nursing home, medications | AP vs none:  HR=0.90(0.75-1.09) |
| Krista 2011 [20] | UK | population-based cohort study | nursing home residents | 1996-2006 | AP users | ≥65 years  (mean: 83-84 years) | 10900 | all-cause mortality (within  180 days) | age, sex, calendar year, level of care and clinical conditions (High-dimensional propensity score) | CAP vs AAP RR=1.52 (1.14–2.02) |
| Nonino 2006 [21] | Italy | population-based cohort | population-based database | 2003 | dementia | ≧65 years  (mean: 82.99 years) | 2314 | all-cause mortality  (90 days) | sex, age, nursing home placement, severity of dementia and functional impairment | AAP vs none  HR=1.023 (0.780–1.342) |
| Trifiro 2007 [22] | Netherlands | nested case-control study | outpatients | 1996–2004 | dementia | ≧65 years | 4421 | all-cause mortality | gender, disease, home-bound lifestyle, medications | 1.AAP vs none:  OR=2.2 (1.2–3.9)  2.CAP vs none:  OR=1.7 (1.3–2.2);  3.CAP vs AAP  OR=0.77(0.42,1.43  4.Past CAP vs none:  OR=1.4 (0.9–2.0);  5.Past AAP vs none:  OR=1.4 (0.4–5.2) |
| Wastila 2009[23] | US | retrospective cohort study | database | 1999-2002 | AP users | unclear | 2363 | all-cause mortality | sex, age, marital status, education, poverty status, urban residence, BMI, physical function, self-reported health, supplemental insurance status, insurance, year, number of reported physical conditions, physical restraint use, disease, medications | 1.CAP vs none:  HR=0.89 (0.67–1.19)  2.AAP vs none:  HR=0.77 (0.62–0.96)  **For dementia**  AP vs none  HR=0.77(0.60–0.98) |
| Huybrechts 2011 [24] | US | cohort study | nursing home patients | 2001–2005 | AP users | ≥65 years  (mean: 82.7-82.8 years) | 82,012 | all-cause mortality  (180 days) | age, sex, race, calendar year, disease, hospitalization, medications | CAP vs AAP  RR=1.71 (1.63-1.79) |
| Suh 2005[25] | UK | prospective cohort study | nursing home patients | unclear | dementia | mean 79.5 years | 273 | all-cause mortality  (1 year) | age, severity of dementia, medical comorbidities, cognitive impairment and behavioral and psychological symptoms of dementia | 1.AP vs none  RR=0.78(0.696,0.882)  2.Alzheimer’s disease  AP vs none  RR=0.776(0.667,0.902) |
| Gisev 2012 [26] | Finland | population-based cohort study | community-dwelling older adults | 2000-2008 | AP users | ≧65 years  (mean: 74.1-76.6 years) | 2224 | all-cause mortality  (9 year) | age, sex, drug use, medications, disease | AP vs none:  HR=2.07 (1.73–2.47) |
| Kales 2007 [27] | US | retrospective cohort study | Veterans affairs registers/ outpatients | 2001-2005 | dementia | ≧65 years  (mean: 79 years) | 10615 | all-cause mortality  (1 year) | age, gender, race, disease, hospital utilization | CAP vs AAP:  RR=1.08(0.86, 1.33) |
| Hartikainen 2005 [28] | Finland | cohort study | database | 1998-2003 | dementia | ≧75 years  (mean: 84 years) | 601 | all-cause mortality | age, sex | AP vs none:  HR=2.75 (1.46–5.19) |
| Liperoti 2009 [29] | US | retrospective cohort | nursing home | 1998-2000 | dementia | ≧65 years  (mean: 83.5-84.5 years) | 9729 | all-cause mortality (6-months) | age, race, gender, BMI, activities of daily living score, cognitive performance scale score, severity of behavioral symptoms, cardiovascular and cerebrovascular comorbidities, medications | 1.CAP vs AAP:  HR=1.26, (1.13-1.42)  2.Alzheimer’s disease  CAP vs AAP:  HR=1.02 (0.75-1.39) |
| Gill 2007 [30] | Canada | population-based, retrospective cohort study | community-dwelling patients | 1997-2003 | dementia | ≧65 years  (mean: 81.4-85 years) | 27,259 | all-cause mortality (30-180 days) | age, sex, and the presence of Charlson comorbidity variables | 1.AAP vs none:  HR=1.32 (1.12–1.54)  2.CAP vs AAP  HR=1.23 (1.00–1.50) |
| Piersanti 2014 [31] | Italy | cohort study | outpatients | 2007-2009 | dementia | ≧65 years | 696 | all-cause mortality  (3 years) | unclear | AAP vs none  RR= 2.354 (1.704-3.279) |
| Raivio 2007 [32] | Finland | prospective cohort Study | geriatric hospitals and nursing homes | 1999-2000 | dementia | mean  86 years | 424 | all-cause mortality  (2 years) | age, gender, disease, comorbidity, functional impairment, medications | 1.CAP vs none:  HR=0.68(0.46-1.03)  2.AAP vs none  HR=0.49(0.24-0.99) |
| Connors 2016 [33] | Australia | cohort study | national institute of health clinical trials registry | unclear | dementia | 78.03±7.5 years | 779 | all-cause mortality  (3 years) | age, sex | 1.CAP vs none  HR=0.66(0.39–1.13)  2.AAP vs none  HR=1.37(1.01–1.85) |
| Jackson 2014 [34] | US | retrospective cohort study | database | 1994-2005 | AP users | ≧65 years | 30941 | all-cause mortality (14-180 days) | demographic characteristics, health services usage, comorbid chronic, psychiatric conditions, and concomitant medication use | CAP vs AAP  RR=1.15(1.08-1.22) |
| Sultana 2014 [35] | UK | cohort study | database | 2007-2010 | vascular dementia | ≧30 years | 1,531 | all-cause mortality (22.7 months) | age at diagnosis of vascular dementia, gender, ethnicity group and cognitive function | AAP vs none  HR=1.05(0.87-1.26) |
| Musicco 2011 [36] | Italy | retrospective population cohort study | computerized health information database | 2002-2008 | alzheimer’s disease | ≧60 years  (78.5 years) | 4369 | all-cause mortality  (2 years) | exposure, sex, comorbidity, medications | 1.CAP vs none:  HR=3.7 (2.6–5.1) 2.AAP vs none:  HR=2.5 (2.0–3.0) 3.CAP vs AAP  HR=1.5 (1.1–2.1) |
| López-Pousa 2006 [37] | US | retrospective cohort study | clinic case registed patients database | 2001-2005 | alzheimer’s disease | 50–92 years  (mean: 78.3-83 years) | 404 | all-cause mortality | age, female , the GDS score, medications, follow-up time | AP vs none:  OR=2.75(1.14–6.65) |
| Arai 2016 [38] | Japan | prospective cohort study | database | 2012-2013 | alzheimer’s disease | mean  81 years | 10079 | all-cause mortality  (24 weeks) | age, sex, body weight, height, in/outpatient, comorbidity, impaired level of dementia, care level. | 1.AP vs none  OR=2.53 (1.04–6.14)  2.Past AP vs none  OR=1.01 (0.72–1.43) |
| Lopez 2013 [39] | US | cohort study | nursing homes residents and outpatients | 1983-2005 | alzheimer’s disease | 72.0±8.6 years | 957 | all-cause mortality (4.3 years) | age, education level, gender, and baseline Mini-Mental State Examination (MMSE) score, disease, medications | 1.CAP vs none  HR=1.30 (0.95–1.79) 2.AAP vs none  HR=1.02 (0.61–1.71) |
| Gardette 2012 [40] | France | prospective cohort study | Community-Dwelling Patients | 2000-2002 | alzheimer’s disease | 77.7±6.8 | 534 | all-cause mortality  (3.5 years) | age, gender, center, history of cardiovascular disease, history of diabetes, MMSE score, ADL score, NPIpsychosis subscore, NPI hyperactivity subscore, denutrition, hospitalization, medical assistance | AP vs none  HR=1.12 (0.59–2.12) |
| Vilalta-Francha 2012[41] | Spain | cohort study | database | unclear | alzheimer’s disease | mean  75 years | 491 | all-cause mortality  (1 year) | age, gender, CAMCOG, general health status, Baseline NPI hallucinations, disease | AP vs none  HR=0.520 (0.121–2.235) |
| Danielsson 2015[42] | Sweden | matched case–control study | patient register database | 2008-2013 | AP users | ≧65 years  (mean: 83.9 years) | 1716552 | all-cause mortality | education, number of contacts with primary care, inpatient days, medications, comorbidities, disease | AP vs none:  OR=2.98 (2.92-3.04) |
| Frandsen 2014 [43] | Denmark | national register-based cohort study | national patient registry database | 1997-2007 | parkinson | ≧20 years | 37256 | all-cause mortality | age, gender, medication discontinuation | 1.CAP vs none:  HR=1.89 ( 1.42, 2.53); 2.AAP vs none:  HR=1.46 (1.20-1.76); |
| Forsaa 2010 [44] | Norway | population-based cohort study | community-based or nursing home | 1993-2005 | parkinson | mean  73.5 years | 230 | all-cause mortality  (16 years) | age, sex, psychiatric disorder, physical illness, drug use | AP vs none:  HR=1.05 (0.70–1.58) |
| Weintraub 2016 [45] | US | retrospective matched-cohort study | veterans health administration database | 1999-2010 | parkinson | 76.3±7.7 years | 15754 | all-cause mortality  (180 days) | age, sex, race, index year, comorabid dementia | 1.AP vs none  HR=2.35, (2.08-2.66)  2..CAP vs none  HR=3.65 (2.47-5.39)  3.AAP vs none  HR=2.26 (1.98-2.57) |
| Ballard 2015[46] | UK | cohort study | database | 2013 | parkinson | 30-80 years  (mean: 71.1 years) | 423 | all-cause mortality  (median: 421 days ) | sex, age, race, BMI, MMSE, disease, CGI severity, duration of PD | AP vs none:  RR=4.20 (2.13-7.96) |
| Marras 2012 [47] | Canada | nested case–control study | healthcare administrative database | 2002-2008 | parkinson | ≧70 years  (mean: 83 years) | 31,328 | all-cause mortality (within 30 days) | Charlson comorbidity index score, dementia, and change in residence (community to long term care) | 1.AAP vs none:  OR=2.0 (1.4–2.7) 2.CAP vs AAP:  OR=2.4 (1.1–5.7) |
| Jackson 2015 [48] | US | cohort study | database | 1994-2005 | AP users | ≧65 years | 26197 | all-cause mortality (180 days) | gender, disease, medications | CAP vs AAP:  RR=1.14, (1.06-1.22) |
| Suvisaari 2013[49] | Finland | cohort study | database | 2000-2001 | AP users | ≧30 years  mean: 54.1 years | 8028 | all-cause mortality  (9 years) | age, sex, diagnostic groups and antipsychotic medication | AP vs none: HR=2.34( 1.86-2.96); |
| Honkola 2012[50] | Finland | case–control study | database | 1998-2009 | AP users | 65.2±11 years | 2732 | sudden cardiac death | gender, history of angina pectoris, hypertension, diabetes, a history of prior AMI, hypercholesterolaemia, and the use of cardiovascular medications | 1.AP vs none: OR=3.44(1.80-6.54)  2.AAP vs none: OR=1.30(0.54-3.11) |
| Kelly 2010[51] | US | retrospective case-control study | administrative database | 1994-2004 | schizophrenia | 20-69 years | 1686 | cardiovascular disease mortality  (6-10 years) | age,race,sex | AP vs none:  HR=1.2 (0.59,2.44) |
| Tiihonen 2009[52] | Finland | population-based cohort study | outpatient patients | 1996-2006 | schizophrenia | unclear | 66881 | 1.all-cause mortality  2. cardiac mortality  (11 years) | sex, age, duration of illness, previous hospital treatment for attempted suicide, disease, and time since start of follow-up | AP vs none  HR=0.68 (0.65-0.71) |
| Tenback 2012[53] | Netherlands | prospective Cohort Study | A health insurer database | 2006-2008 | schizophrenia | unclear | 105,141 | all-cause mortality | age, sex, and medications | 1.AP vs none  HR=2.6 (2.0-3.2)  2.CAP vs none  HR=2.36 (1.38-4.04) 3.AAP vs none  HR=0.89 (0.48-1.64) |
| Kiviniemi 2013[54] | Finland | prospective register-based cohort study | national registers database | 1998-2003 | schizophrenia | 16-65 years | 6987 | 1.all-cause mortality  2.cardiac mortality  (5 years) | age, gender, comorbid physical diseases and patient group, | **All-cause mortality** 1.CAP vs none:  OR=1.76 (1.28, 2.42) 2.AAP vs none:  OR=0.61 (0.46, 0.79)  **Cardiacmortality**  CAP vs none:  OR=2.00 (1.11, 3.61) AAP vs none:  OR=0.79 (0.45, 1.39) AP vs none:  OR=0.61 (0.25, 1.52) |
| Haukka 2008 [55] | Finland | cohort study | hospitalised patients | 1997-2003 | schizophrenia | ≧16 years  (median: 35.63- 41.05 years) | 1611 | all-cause mortality  (4.3 years) | sex, medications | 1.AP vs none  RR= 0.55 (0.32–0.93),  2.Past AP vs none  RR= 1.12 (0.70–2.05), |
| Enger 2004 [56] | US | case control study | ingenix research database | 1995-1999 | schizophrenia | 38.2±14.0 | 11520 | 1.all-cause mortality  2.cardiac mortality  (1.76 years) | age, sex, year of index dispensing, history of diabetes, medications | **All cause mortality** 1.AP vs none  RR=4.41(2.96–7.91);  2.CAP vs none  RR=4.59 (1.67–12.58); 3.AAP vs none  RR= 4.35 (0.97–19.62);  **Cardiacmortality**  AP vs None  RR=1.30 (0.25–6.65) |
| Baandrup 2010 [57] | Denmark | population-based nested case-control study | register database | 1996-2005 | schizophrenia | 18-53 years | 2130 | all-cause mortality (90 days) | all antipsychotic and somatic comedication categories,epilepsy, and benzodiazepines | AP vs none  OR=0.68(0.41,1.12) |
| Hou 2015 [58] | Taiwan | case control study | database | 1985-2008 | schizophrenia | mean  45 years | 153 | sudden cardiac death | gender, marriage, Living with family, Education, employment, socio-economic status | CAP vs none  RR=5.13(1.61–16.40) |
| Chen 2015 [59] | Taiwan | nationwide population-based cohort study | national health insurance database | 1998-2008 | schizophrenia | ≧10 years  (38.34-38.6years) | 1624 | sudden cardiac death  (3.81-3.91 years) | gender, age, residence, insurance premium, charlson comorbidity index, hospital admission days, and hospital admission times | CAP vs AAP  HR=1.72(1.04,2.94) |
| Tiihonen 2011[60] | Finland | cohort Study | national database | 2000-2007 | schizophrenia | 16-65 years  (mean: 37.8 years) | 2588 | all-cause mortality  (mean: 2 years) | sociodemographic and clinicalvariables, the temporal sequence of the antipsychotics used, and the choice of the initial antipsychotic | AP vs none  HR=0.45 (0.31–0.67) |
| Murray-Thomas 2013 [61] | UK | case control study | general practice research database | 1997-2001 | schizophrenia | 60.3±22.1 years | 922038 | 1.all-cause mortality;  2.cardiac death  (mean: 4 years) | age, sex, socioeconomic status, smoking history, alcohol use, and body mass index (BMI), medications. | **All-cause mortality**  CAP vs AAP  RR=1.2(1.18,1.25)  **Cardiac death:**  CAP vs AAP  RR=1.12(1.03,1.22) |
| Prior 2014 [62] | Denmark | population-based cohort study | health care database | 2003-2010 | stroke | ≧18 years  (mean: 71.77-72.40 years) | 81143 | post-stroke mortality (30 days) | year of admission, severity of stroke, and modified charlson’s index | 1. AP vs none  RR=1.42 (1.29–1.55)  2.Past APvs none  RR=1.05(0.98–1.14) |
| Wang 2014 [63] | Taiwan | nested case–control | national health research institute database | 2010-2011 | stroke | ≧18 years  (mean: 76-77 years) | 47225 | acute ischemic stroke mortality (30 days) | patient gender, age, disease, length of stay and admitted to ICU, current use, dose, and past use of antipsychotics | 1.AP vs none:  OR=0.13 (0.1–0.16)  2.CAP vs none:  OR=0.22, (0.18-0.26)  3.AAP vs none:  OR=0.14 (0.12-0.17) |
| Leece 2015 [64] | Canada | population-based, nested case-control study | database | 1994-2010 | use Methadone Therapy | 31-48 years  (median: 39-42 years) | 1048 | opioid-related death | medication exposures, baseline characteristics, health service utilization, comorbidities | AP vs none  OR=2.32 (1.54-3.48) |
| Acharya 2013 [65] | US | retrospective cohort study | database | 2009-2010 | depression | mean  61 years | 1428 | all-cause mortality | demographics, concomitant drugs, and comorbidities |  |
| Nilsson 2002 [66] | Sweden | case control study | inpatient | 1980-1989 | epilepsy | 17-75 years | 6880 | all-cause mortality | age, sex, occurrence of psychiatric illness and alcoholism | AP vs none:  RR=3.8 (1.0–13.7) |
| Christiansen 2008 [67] | Denmark | population-based cohort study | inpatient | 1991-2004 | peptic ulcer perforation | unclear | 2033 | all-cause mortality  (30 days) | age, gender, level of comorbidity, alcoholism-related diseases, previous uncomplicated peptic ulcer, psychiatric disease, use of anti-ulcer drugs, use of ulcer-inducing drugs and use of antidepressants | 1. AP vs none:  RR=1.7 (1.2–2.3)  2.CAP vs none:  RR=1.6 (1.1–2.2)  3.AAP vs none:  RR=2.1 (0.9–5.1)  4.Past AP vs none:  RR=1.0 (0.7–1.3)  4.Past CAP vs none  RR=1.0 (0.7–1.3) 5.Past AAP vs none:  RR=0.8 (0.2–3.4) |
| Barnett 2006 [68] | US | cohort study | inpatient or outpatient | 2003 | pneumonia | 69.2±12.4 years | 14,057 | in-hospital mortality  (180 days) | age, gender, marital status, race, VA service connection, comorbidity, admission source, bacterium pathology, mental health diagnoses, and inpatient facility. | 1.CAP vs none:  OR=1.51 (1.04–2.19); 2.AAP vs none:  OR=1.20 (0.96–1.50) |
