# Supplementary material for: Does antipsychotic drug use increase the risk of long term mortality? A systematic review and meta-analysis of observational studies
Source: Oncotarget. 2018 Jan 10;9(19):15101–10. doi: 10.18632/oncotarget.24120 (PMC5871101; doi:10.18632/oncotarget.24120)
Supplement: Supplementary file 3 [file oncotarget-09-15101-s003.docx]

**Supplementary table 2. Risk of bias in included studies**

| **Reference** | **Study Selection (Max 4*)** | **Comparability (Max 2*)** | **Exposure**  **(Max 3*)** |
| --- | --- | --- | --- |
| Straus 2004 [1] | ******** | ***** | ******* |
| Ray 2001 [2] | ******** | ****** | ****** |
| Ray 2009 [3] | ******** | ****** | ****** |
| Gisev 2011 [4] | ******** | ****** | ******* |
| Rajender 2012 [5] | ******** | ***** | ****** |
| Wang 2005 [6] | ******** | ****** | ****** |
| Murray-Thomas 2013 [7] | ******** | ****** | ******* |
| Jolly 2009 [8] | ******** | ***** | ******* |
| Sikirica 2014 [9] | ******** | ****** | ****** |
| Nordon 2009 [10] | ******** | ***** | ******* |
| Hwang 2014 [11] | ******** | ***** | ****** |
| Mace 2015 [12] | ******** | ****** | ****** |
| Park 2015 [13] | ******** | ***** | ****** |
| Setoguchi 2008 [14] | ******** | ****** | ****** |
| Schneeweiss 2007 [15] | ******** | ****** | ****** |
| Pratt 2010 [16] | ******** | ***** | ****** |
| Elie 2009 [17] | ******** | ***** | ******* |
| Naksuk 2015 [18] | ******** | ****** | ****** |
| Selbæk 2016 [19] | ******** | ****** | ******* |
| Krista 2011 [20] | ******** | ****** | ****** |
| Nonino 2006 [21] | ******** | ***** | ****** |
| Trifiro 2007 [22] | ******** | ****** | ******* |
| Wastila 2009[23] | ******** | ****** | ****** |
| Huybrechts 2011 [24] | ******** | ****** | ****** |
| Suh 2005[25] | ******** | ****** | ****** |
| Gisev 2012 [26] | ******** | ****** | ******* |
| Kales 2007 [27] | ******** | ***** | ****** |
| Hartikainen 2005 [28] | ******** | ***** | ******* |
| Liperoti 2009 [29] | ******** | ****** | ****** |
| Gill 2007 [30] | ******** | ****** | ****** |
| Piersanti 2014 [31] | ******** | NA | ******* |
| Raivio 2007 [32] | ******** | ***** | ******* |
| Connors 2016 [33] | ******** | ***** | ******* |
| Jackson 2014 [34] | ******** | ****** | ****** |
| Sultana 2014 [35] | ******** | ***** | ****** |
| Musicco 2011 [36] | ******** | ***** | ****** |
| López-Pousa 2006 [37] | ******** | ***** | ****** |
| Arai 2016 [38] | ******** | ****** | ****** |
| Lopez 2013 [39] | ******** | ****** | ******* |
| Gardette 2012 [40] | ******** | ****** | ****** |
| Vilalta-Francha 2012[41] | ******** | ***** | ****** |
| Danielsson 2015[42] | ******** | ***** | ******* |
| Frandsen 2014 [43] | ******** | ***** | ****** |
| Forsaa 2010 [44] | ******** | ****** | ******* |
| Weintraub 2016 [45] | ******** | ***** | ****** |
| Ballard 2015[46] | ******** | ***** | ****** |
| Marras 2012 [47] | ******** | ***** | ******* |
| Jackson 2015 [48] | ******** | ****** | ****** |
| Suvisaari 2013[49] | ******** | ****** | ******* |
| Honkola 2012[50] | ******** | ***** | ******* |
| Kelly 2010[51] | ******** | ****** | ******* |
| Tiihonen 2009[52] | ******** | ****** | ******* |
| Tenback 2012[53] | ******** | ***** | ******* |
| Kiviniemi 2013[54] | ******** | ****** | ******* |
| Haukka 2008 [55] | ******** | ***** | ******* |
| Enger 2004 [56] | ******** | ****** | ******* |
| Baandrup 2010 [57] | ******** | ***** | ******* |
| Hou 2015 [58] | ******** | ***** | ******* |
| Chen 2015 [59] | ******** | ****** | ******* |
| Tiihonen 2011[60] | ******** | ****** | ******* |
| Murray-Thomas 2013 [61] | ******** | ***** | ******* |
| Prior 2014 [62] | ******** | ***** | ****** |
| Wang 2014 [63] | ******** | ****** | ******* |
| Leece 2015 [64] | ******** | ****** | ******* |
| Acharya 2013 [65] | ******** | ***** | ***** |
| Nilsson 2002 [66] | ******** | ****** | ******* |
| Christiansen 2008 [67] | ******** | ****** | ****** |
| Barnett 2006 [68] | ******** | ****** | ****** |
